# Explainable AI-Based Intrusion Detection Systems for IoT Environments: A Systematic Literature Review

**DOI:** 10.3390/s26185744

**Published:** 2026-09-10

**Authors:** Murat Varol, Aykut Karakaya

**Affiliations:** Department of Computer Engineering, Zonguldak Bülent Ecevit University, Zonguldak 67100, Türkiye; aykut.karakaya@beun.edu.tr

**Keywords:** IoT, lightweight, XAI, IDS

## Abstract

With the rapid proliferation of Internet of Things (IoT) technology today, the number of devices connected to each other through these networks is increasing significantly, and the security risks these devices face from cyberattacks have emerged as a clear problem. Intrusion detection systems (IDSs) are emerging as a solution to this problem and play a significant role in securing IoT-based networks. Although machine learning and deep learning-based IDS approaches have achieved high accuracy rates in detecting attacks in recent years, the lack of transparency in these models’ decision-making processes poses a major drawback in terms of reliability and explainability. To ensure that the decisions of IDS models are understandable and to address this issue, Explainable AI (XAI) approaches are being implemented. This study provides a detailed review of the current literature on XAI-enabled IDSs developed for IoT environments. The studies examined are systematically evaluated in terms of the deep learning models used, lightweight model designs, explainability methods and validations, approaches to protecting data privacy, and datasets. The literature review highlights that research is not only focused on the accuracy of attack detection but also aims to design IDS solutions that are lightweight, explainable, and privacy-preserving. This paper aims to provide a foundational resource to guide future research on reliable, explainable, and practical IoT intrusion detection systems by identifying the problems addressed in current research and highlighting the limitations in the literature. In addition, this work proposes and applies a study assessment framework to systematically assess methodological adequacy, experimental reproducibility, lightweight deployment, XAI techniques, and XAI validation.

## 1. Introduction

The Industry 4.0 transformation has brought the concept of the Internet of Things (IoT) to the forefront, accelerated the digitization of industrial systems, and highlighted the importance of integrating Operational Technology (OT) with Information Technology (IT). IoT and Industrial Internet of Things (IIoT), the core components of this transformation, refer to the continuous interconnection and collaboration of sensors, actuators, programmable logic controllers (PLCs), smart devices, and communication infrastructure. Thanks to IIoT technologies, critical functions such as real-time monitoring, process optimization, preventive maintenance, and data-driven decision-making have been integrated into today’s technology. However, the increasing number of interconnected devices and their growing access to the internet have made IoT and IIoT systems more vulnerable to cyberattacks.

Unlike IT systems, OT and IIoT components must meet the needs of high availability, reliability, and real-time operation. Failure to meet these needs, i.e., cyberattacks targeting systems, can lead to data loss, disruption of production processes, equipment damage, and threats to human safety. The increasing number of cyberattacks targeting critical infrastructure in recent years has highlighted the need for research addressing the security of IoT and IIoT systems.

In this context, intrusion detection systems (IDSs) have become one of the security solutions used to detect anomalous packets and malicious attempts in network traffic, and have been the subject of much research in recent years. The adaptation of artificial intelligence and machine learning algorithms to IDSs has enabled high success rates in detecting previously undefined attack types. The inclusion of deep learning (DL) techniques in IDSs has also yielded significant results in many current studies in the field of cybersecurity.

Additionally, a large portion of AI-based IDS models produce “black box” results. This makes it difficult to understand how a particular attack was detected or what characteristics were used to make a decision. In industrial critical infrastructure environments, it is crucial for operators and cybersecurity professionals to be able to verify and trust the results produced by AI-powered models. This is where explainable artificial intelligence (XAI) techniques have emerged, increasing researchers’ interest in this area. XAI techniques detail the decision-making processes of AI models, making them more transparent and interpretable, thus offering important features such as reliability and accountability [1].

On the other hand, devices in the IIoT ecosystem have low hardware specifications and are subject to limitations such as limited processing power, low memory capacity, and low storage space. Running artificial intelligence and XAI algorithms on resource-constrained industrial system components such as embedded systems, sensors, and PLCs is beyond the capabilities of these devices and leads to performance issues. Therefore, research on lightweight machine learning algorithms and lightweight XAI models has intensified in recent years.

Edge computing emerged to help address the problem of resource-constrained devices. This approach is based on the logic of processing data on computers closest to the data source, rather than sending data generated on resource-constrained devices to powerful, resource-unconstrained cloud systems on remote servers. This reduces latency in the data processing process and minimizes bandwidth usage by decreasing internet usage. Consequently, it also enables the real-time detection of threats. However, implementing and running IDS and XAI techniques in edge environments presents an optimization problem between limited resources and explainable high accuracy. This situation has triggered the development of lightweight and explainable IDS models, making them a significant focus of current research.

Although numerous studies exist in the literature on IoT and IIoT security, AI-based IDS models, lightweight frameworks, and XAI techniques, the number of studies that examine lightweight and explainable IDS solutions at the intersection of these fields from a holistic perspective remains limited. Many AI-based IDS models offering high accuracy rates lack explainability and present various application challenges in resource-constrained industrial devices. While most current review studies focus on specific topics, there is a need for comprehensive, up-to-date reviews that evaluate and improve the applicability of XAI-supported lightweight IDS solutions usable in resource-constrained industrial environments. Studies published particularly in the last two years have focused on the integration of XAI and lightweight framework methods into IDSs.

The aim of this study is to present a comprehensive systematic literature review of IDSs used in IoT and IIoT environments, examining them from the perspectives of explainable artificial intelligence, lightweight approaches, and resource-constrained devices, using studies published from 2024 onwards. To this end, an in-depth examination of current research and a comparative evaluation of the datasets, machine learning models, deep learning models, XAI techniques, and lightweight IDS approaches used is critical for designing reliable, explainable, and resource-efficient IDS solutions for the future. This study aims to fill this research gap by holistically addressing explainable, lightweight, and implementable IDS approaches that can be used in IoT and IIoT environments. Accordingly, existing studies are analyzed, current methods and their results are compared, and potential directions for future research are discussed.

### 1.1. Research Questions

This systematic literature review aims to address and answer the following research questions:

- According to the study assessment framework we have defined, to what extent do the included studies meet the criteria for methodological quality, experimental reproducibility, lightness and XAI evaluation?
- What are the current trends and limitations of the lightweight design methods employed in the development of new intrusion detection systems for Internet of Things (IoT) environments?
- To what extent does the literature meet the trends and validation needs of XAI techniques used to interpret the results produced by lightweight attack systems?

### 1.2. Contributions

The contributions of this study are as follows:

- This study comprehensively examines XAI-supported intrusion detection systems developed for IoT, IIoT, and various IoT environments, focusing on current literature.
- In the literature, intrusion detection system solutions are examined comparatively in terms of data preprocessing, feature selection, model architecture, class imbalance problems, and explainability concepts.
- Studies conducted on lightweight IDS approaches that can run on resource-constrained devices and high-performance deep learning techniques are compared.
- By identifying the common focuses, strengths, and shortcomings of existing studies, research gaps are determined in areas such as datasets, explainability, generalizability assessments, and applicability in real-time network environments.
- Information that will guide future research on adaptive learning, explainability, lightweight model design, data privacy, and real-time applications of IDSs is provided.
- This study goes beyond simply summarizing existing information in the literature; it also critically analyzes each study through a detailed evaluation, determining whether the proposed approach is lightweight, its strengths and weaknesses, and its limitations.
- A unique study assessment framework is developed to assess the methodology, experimental reproducibility, lightweight status, and XAI capabilities of the studies under review.

### 1.3. Organization

This study consists of six sections. Section 1 presents the scope, purpose, and motivation of the study. Section 2 provides information about the fundamental concepts within the scope of the research. Section 3 describes the research methodology used in this study in detail. Section 4 examines and comparatively evaluates the studies selected in accordance with the methodology. Section 5 discusses the results obtained and interprets the findings, supported by graphs. Finally, the overall conclusions of the study are summarized, and suggestions for future research are provided.

## 2. Conceptual Framework

This section provides general information about the concepts covered in this study. The concepts of IoT, IIoT, IDS, and XAI are explained in separate subsections, and prominent studies in these fields are briefly mentioned. The relationships between these concepts are also presented in this section.

### 2.1. IoT

The IoT refers to a communication network that enables devices and the systems they are connected to to connect to the internet via sensors, actuators, and software, and to share and process data with larger computer networks. In the early days of the internet, the vast majority of data was manually generated and processed by humans. With the IoT, data collection can be carried out autonomously and flawlessly without human intervention. In this case, accurate data collection is a crucial step in instantly detecting cyber threats. The growing IoT ecosystem [2,3], with billions of devices connected to this communication network, increases the penetration surface for attackers. Therefore, the problem arises of whether security monitoring can be performed accurately and quickly by humans. To solve this problem, AI-powered intrusion detection systems (IDSs) are being developed.

Intrusion detection systems automatically collect log data generated by IoT devices. By using this data, IoT devices can predict physical machine failures, reducing maintenance costs and system downtime. IDSs can quickly detect anomalies and abnormal data flows that might escape human control using artificial intelligence [4,5,6,7]. The system is based on the principle that devices can make decisions and take security measures on their own without human intervention. For example, when a cyberattack or malware is detected on a smart device, the infected IoT device is automatically isolated from the network. This prevents the attack from spreading to all of the company’s computers without human intervention.

With the miniaturization of processors, the decrease in the cost of sensors, and the IPv6 infrastructure, every object is now connected to the internet. This situation has both advantages and disadvantages. Thanks to devices communicating with each other without human intervention, the remote control of boilers, air conditioners, refrigerators, washing machines, TVs, and tea/coffee makers is possible, ensuring beverages are ready at desired times. Another advantage is efficiency; in smart city systems [8,9], streetlights can be turned on only when needed, boilers can operate according to demand, and irrigation systems can function based on soil moisture levels, preventing electricity and water waste. Looking at the use of IoT in healthcare systems [10,11,12], it enables the 24/7 transmission of patient information, such as pacemaker status, blood sugar, and blood pressure measurements, to doctors for individuals who require real-time health monitoring. When medical intervention is needed, an ambulance is automatically called, ensuring the patient is quickly transported to hospital.

The IoT is also important for resource-constrained systems. These systems require lightweight protocols and cryptographic frameworks to ensure security [13,14,15]. Potential limitations for IoT applications, such as smart home systems, connected vehicles, health monitoring systems, and smart cities, lie in the ability of resource-constrained systems to ensure secure communication under limited processing power, memory capacity, and energy resources. Combining lightweight and explainable security approaches can contribute to the development of more reliable and viable systems in resource-constrained IoT environments.

On the downside, the fact that every object is connected to the internet means a new attack surface for cyber attackers. Smart TVs, ovens, robot vacuum cleaners, and smart children’s toys can be hacked, allowing access to the entire home network. The uninterrupted monitoring of all data, the learning of habits and location information, and a smart bed tracking sleep times can all lead to data privacy breaches. Every internet-connected device may have an outdated software version containing security vulnerabilities. Therefore, these devices need to receive software updates; otherwise, they become easy targets for cyber attackers. Devices collect data and make automated decisions, but managing these systems and making strategic decisions always requires expert human knowledge.

The IIoT is the adapted version of IoT technology for devices in industrial sectors such as manufacturing, logistics, agriculture, and oil and gas. The IIoT creates a large industrial network by connecting machines, robots, production lines, and plant infrastructure in factories to the internet via sensors. While IoT devices focus on making life easier, the IIoT helps increase operational efficiency, reduce costs, and ensure workplace safety. Industrial sensors are devices mounted on machines that measure data such as temperature, vibration, and pressure. Edge computing is the real-time processing of critical factory data on a local computer closest to the machine, without sending it to cloud systems. Data is transmitted over highly secure wired (Ethernet, PROFINET) or wireless infrastructures that are robust enough to withstand the harsh conditions of factories. Network TAP devices are placed in between to listen to and record data in the network environment [16]. This collected data is analyzed using centralized software such as SCADA or AI-based cloud platforms, and reporting is then performed.

Connecting industrial facilities to the internet creates an opportunity for cyber attackers to threaten critical infrastructure such as nuclear power plants and energy grids. The Stuxnet attack in 2010 is the best-known example of this. For this reason, security measures in IIoT systems are at the highest level. Even the slightest anomaly in factory data is detected by threat detection systems, and the entire communication line is secured within seconds. Research on IoT security encompasses a variety of methods [17,18,19].

Zero Trust is a cybersecurity strategy that, unlike traditional network security approaches, argues that no user, device, or system should be trusted, regardless of whether it is inside or outside the network. It is an approach that requires verification every time a user accesses the network. This approach is broken down into three main principles according to NIST standards: Explicitly Verify, Least Privilege Access, and Assume Breach. Explicitly Verify verifies the user’s identity, location, and the service or data class they are using at every access request. Least Privilege Access grants users and devices like IoT only the access they need at that moment to perform their tasks. Assume Breach assumes that the system could be hacked at any time or that cyber attackers could be inside; therefore, networks are divided into small segments and continuous analysis is performed for anomaly detection. Recent studies addressing Zero Trust architecture have discussed implementation challenges [20] and proposed a DDoS security solution for 5G-based IoT systems [21].

### 2.2. IDSs

With the acceleration of digital transformation, the cyber threat surface faced by companies is increasing. Traditional firewalls, like a gatekeeper at the network’s entry point, only monitor traffic that adheres to certain rules. However, traditional firewalls are insufficient to catch a malicious attacker who can access the inner workings of the network, and instead, IDSs are used. An IDS is a security solution that monitors and detects malicious activity, policy violations, and cyber threats on a computer network or system. While firewall devices control network traffic at entry ports, an IDS acts like an alarm system that monitors suspicious activity that has managed to infiltrate the system. IDSs use two different methods to detect threats: signature-based and anomaly-based. In the signature-based method, a database containing signatures of previously known cyberattacks is used. When traffic on the network matches these signatures, the system issues an alert. However, it is ineffective against newly emerging and as yet unidentified attacks. In the anomaly-based method, artificial intelligence algorithms are used to create a profile of the normal behavior of traffic on the network. Any unusual behavior that deviates from this normal profile is flagged as an anomaly, and the system issues a warning. Because it is behavior-based, it is effective against zero-day attacks. SNORT and Suricata are two of the most popular open-source software programs used in the cybersecurity world to monitor network traffic, detect malicious activity, and prevent cyberattacks [22].

Among the biggest challenges for IDSs are false positives, scalability issues, and a lack of adaptation to new attack types. These factors directly affect the reliability and effectiveness of the systems, highlighting them as critical points requiring continuous improvement in academic studies. IDSs can often mistake normal network traffic for an attack. This can mislead system administrators and lead to the overlooking of genuine attack traffic. Maintaining IDS performance in large and complex networks is difficult because increased network traffic can exceed the system’s analytical capacity, causing delays and security vulnerabilities. Since attackers are constantly developing new methods, signature-based IDSs may be insufficient in identifying these attacks. Zero-day attacks, in particular, pose a major problem for IDSs. IDSs require high processing power and memory, and performance issues occur on devices with low hardware specifications. The continuous monitoring of network traffic by IDSs can raise concerns regarding user privacy.

### 2.3. XAI

XAI is a field of artificial intelligence that aims to justify the decisions, predictions, and behaviors of AI and machine learning models in a way that is understandable, transparent, and logical to humans. Traditional AI models produce output from input, but XAI includes information on why the output was produced based on the given input. In short, XAI aims to open the black box and transform it into a transparent, accountable white box. XAI is considered a necessity, especially in critical sectors where the margin of error can lead to human loss or significant financial loss (healthcare, banking, autonomous vehicles, cybersecurity). For example, when a loan application is rejected by AI, legal regulations require a transparent explanation of the reason for rejection (insufficient income, past payment irregularities, etc.). Another example is when an autonomous vehicle suddenly brakes; engineers need to know whether the system perceived a roadside object as a threat or whether it was due to a software error. When an intrusion detection system flags and blocks traffic on the network as “malicious”, it must explain to the security analyst what anomaly it based this decision on.

For XAI, data scientists use specific mathematical and visual techniques: SHAP, LIME, and heatmaps [23]. SHAP and LIME are popular algorithms that break down the decisions of complex machine learning models into local components, showing how much each feature influences the outcome. Heatmaps, especially in visual processing models, are analytical tools that use colors to show exactly which pixels of an image the AI is focusing on when making a decision.

The integration of XAI and IoT creates an ecosystem that automates the data flow generated by billions of smart devices, making the logic behind decisions transparent and auditable. When traditional AI models predict a failure or automatically open a valve, operators cannot see why that decision was made. This can create significant risks in terms of cybersecurity and occupational health. When XAI is implemented, sensors in an industrial IoT network provide explanations in human language within seconds of when a critical system is stopped, such as “I stopped production due to a 25% increase in motor temperature and a vibration anomaly in the gearbox”. This integration makes it easier to understand whether IoT networks making critical decisions, from smart cities to autonomous vehicles, have been subjected to a cyberattack or experienced a real physical failure. This maximizes both the operational safety of the systems and their reliability among people.

The combined use of XAI and IDSs [24,25,26] provides a security architecture that makes AI-generated alarms completely transparent, understandable, and verifiable for cybersecurity analysts in modern cyber defense. Traditional machine learning-based anomaly detection systems (black box) cannot explain to analysts whether a movement in network traffic is a cyberattack or harmless network congestion when it signals an alarm as “malicious”; this leads to significant time losses and false positives in operation centers (SOCs). However, when XAI is integrated into IDSs, it provides mathematical and logical justifications for its decision when blocking an IP address, such as “I marked this traffic as malicious because the packet sizes are four times larger than normal”. This powerful combination, which builds a bridge of trust between humans and artificial intelligence in threat hunting, enables security teams to identify the root cause of the threat in seconds and make the most accurate intervention, especially in complex corporate networks where cyber attackers use advanced methods.

In XAI, deep learning (DL) approaches or hybrid models can be used just as classical ML is. Thanks to its multi-layered structure, deep learning can automatically learn complex and nonlinear relationships in the data. However, as model depth increases, interpreting decision processes can become challenging. Methods such as CNNs and LSTMs can produce highly accurate models in IDSs. In addition to standard XAI methods like SHAP and LIME, deep learning-based mechanisms such as attention mechanisms and class activation mapping also support explainability.

**Figure 1:**
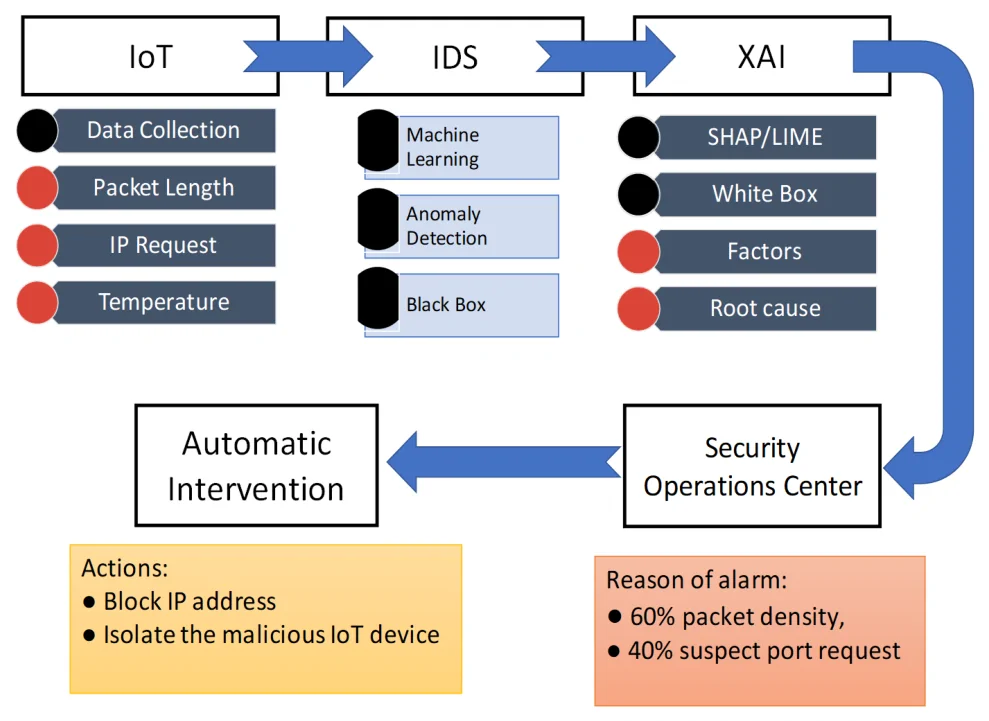
General diagram of concept paradigms.

Figure 1 illustrates the cycle processes of the IoT, IDS, and XAI within a company. The IoT is used for data collection and transmits this data to the IDS. Based on the received data, the IDS uses artificial intelligence to detect anomalies. However, XAI comes into play to determine the cause of this detection. The transformation from a black box to a white box is accomplished through XAI. This cause is then reported to the SOC (security operations center) office and operators to understand the reason for the anomaly. Based on this anomaly, automated intervention processes are activated, blocking the IP address of the suspicious device and isolating it from the network. By analyzing the cause of the anomaly, it can be determined whether the suspicious device is truly the result of a cyberattack or a malfunction.

## 3. Research Methodology

This paper is structured to systematically review the recent studies in the literature on explainable and lightweight intrusion detection systems in IoT networks. The scope of the research focuses on IDSs operating in IoT environments that have low resource consumption and incorporate XAI approaches. Accordingly, the literature review was conducted across IEEE Xplore, ScienceDirect (Elsevier), SpringerLink, MDPI, and Wiley, with the final database search performed on 16 April 2026. At this stage, the following advanced search expression was used: (“Internet of Things” OR IoT OR IIoT) AND (“intrusion detection” OR “intrusion detection system” OR IDS) AND (“explainable artificial intelligence” OR XAI OR explainability OR explainable) AND (lightweight OR “resource-constrained” OR “resource constrained” OR “edge computing”).

**Figure 2:**
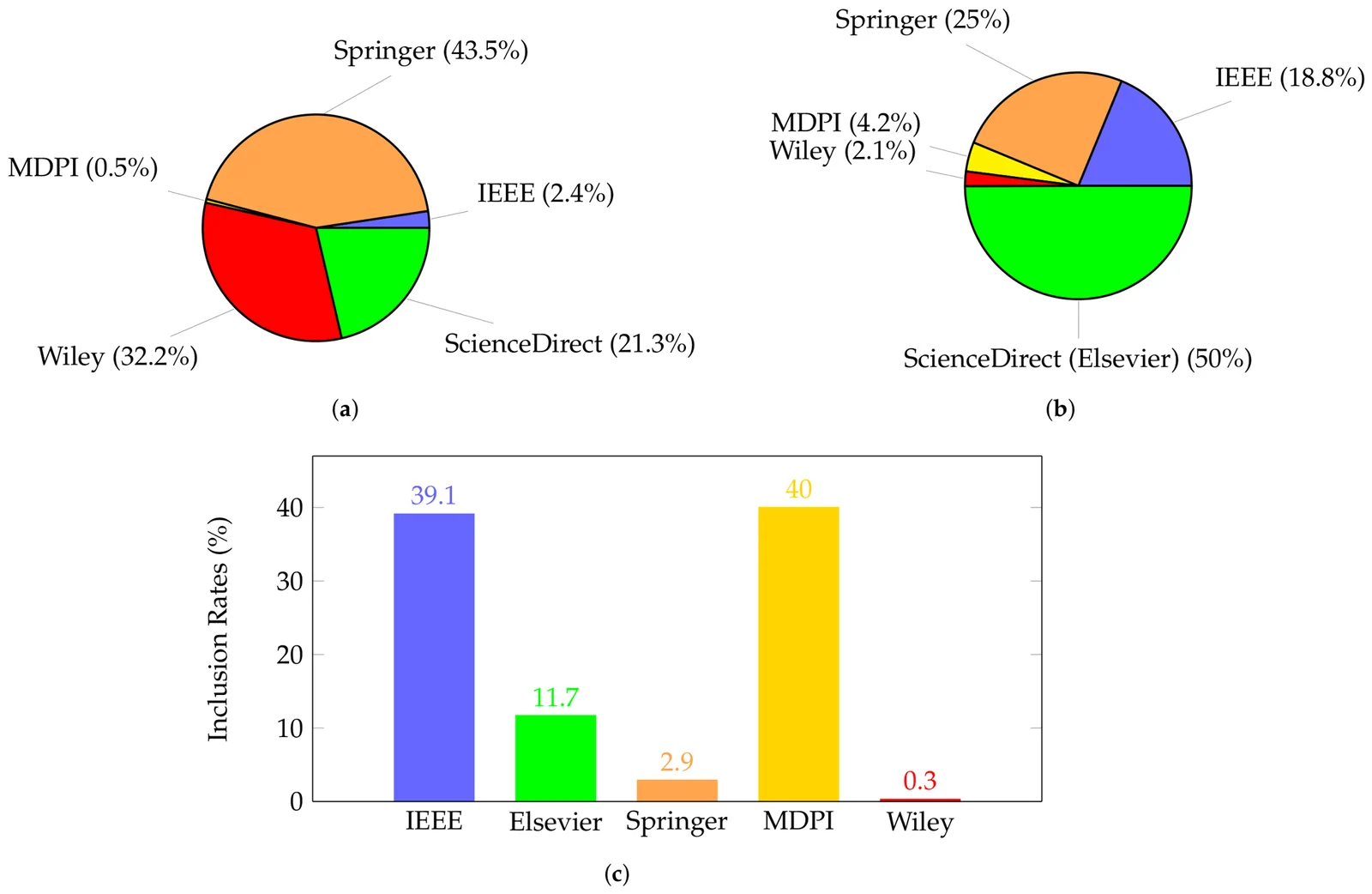
Distribution of the papers retrieved using the advanced search string across databases. (**a**) Initial advanced search results. (**b**) Results after applying the filtering, inclusion, and exclusion criteria. (**c**) Advanced search hit rates.

All database searches were conducted in English only and included research studies published from 2024 to the specified date. This study was not preregistered and no review protocol was registered in a public registry. When searching the IEEE database, the “Journals” option was selected as a filter. When searching the ScienceDirect database, since the length of the given search expression exceeded the specified limit, abbreviations of the expressions were used in the search. When searching the Springer database, the expressions “article” and “computer science” were selected as filters. When searching the Wiley database, the expressions “journal” and “computer science” were selected as filters. In the MDPI database, the search was performed without filtering. After all these filters, a total of 965 records were obtained: 23 records in the IEEE database, 206 in the ScienceDirect database, 420 in the Springer database, 5 in the MDPI database, and 311 in the Wiley database. This distribution is shown in Figure 2a. All retrieved records from IEEE, Springer, ScienceDirect, Wiley, and MDPI were manually examined for duplicate entries before the screening process.

Studies obtained from the advanced search results were pre-evaluated in terms of title, abstract, keywords, and scope of study. Then, 526 articles that were not directly related to source constraints, lightweight model design, IoT, IDSs, or XAI were excluded. The main topics addressed in this paper were used as the selection criteria for the studies identified. The authors jointly screened the titles and abstracts of the identified studies and subsequently assessed the full texts of potentially eligible articles. During the screening and eligibility assessment stages, any differences in interpretation or study selection were discussed between the authors until a consensus was reached. Thus, the screening, eligibility assessment, and review processes were conducted collaboratively by the authors through discussion and consensus. No automation tools were used during the study selection or review process.

Furthermore, since this study was intended to be based on current literature with a high impact factor, 391 studies that were not published in any of the journals in the Q1 or Q2 category of the Journal Citation Reports (JCR) 2025 edition were eliminated from the remaining 439 studies after the exclusion process.

**Figure 3:**
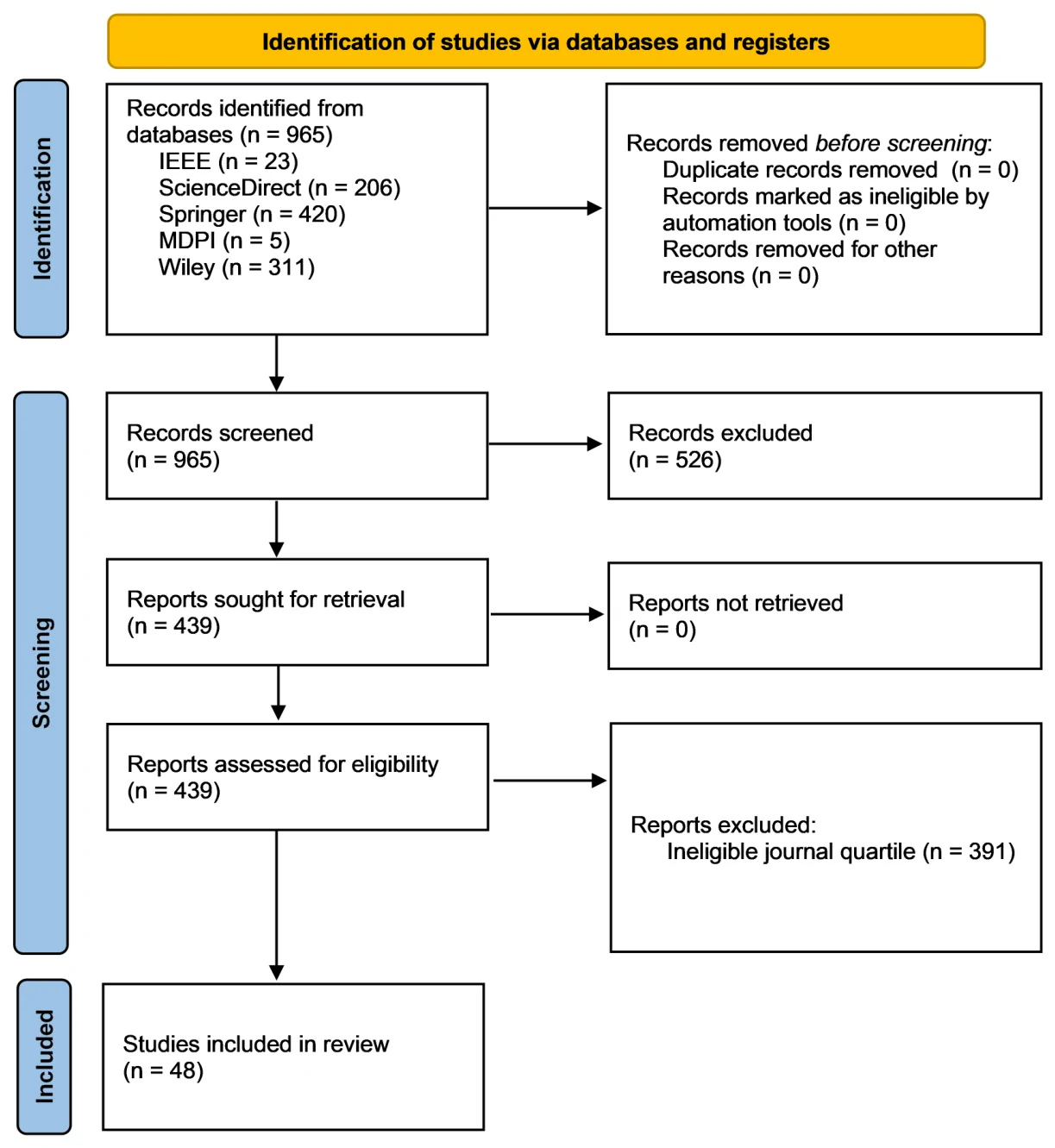
PRISMA diagram of this SLR.

A total of 48 articles are included in the detailed review, considering both relevance to the topic and publication quality. The distribution of the selected studies according to databases is as follows: 9 in IEEE, 24 in ScienceDirect, 12 in Springer, 2 in MDPI, and 1 in Wiley. The distribution is shown in Figure 2b. The suitability of the search strategy, based on the advanced search results and the results obtained after applying the selection criteria, is presented in Figure 2c. The PRISMA flow diagram, encompassing the entire advanced search and the inclusion and exclusion processes, is presented in detail in Figure 3.

In conclusion, the 48 selected articles are systematically reviewed in terms of the ML and DL methods used, explainability techniques, lightweight model designs, edge and IoT integration levels, performance metrics and results, strengths, and limitations. This systematic review was registered in the Open Science Framework (OSF) during the revision stage. The registration is available online: https://doi.org/10.17605/OSF.IO/CEHRW. PRISMA 2020 Checklist available in Supplementary Table S1.

### Study Assessment Framework

A study assessment framework has been developed to systematically characterize the methodological diversity, experimental scope, explainability, lightweight status, and practical applicability of the studies examined. This framework aims not to classify studies as scientifically valid or invalid, but to quantify the extent to which fundamental methodological and reporting features related to lightweight IoT attack detection and explainability are addressed. No formal risk-of-bias or scientific quality assessment was performed. Instead, the included studies were systematically evaluated using the study assessment framework to characterize their reported methodological and experimental features, strengths, and limitations. Equations (1) and (2) provide an overall score for this framework.

(1) $$S_{i}=M_{i}+D_{i}+H_{i}+So_{i}+E_{i}+L_{i}+X_{i}+V_{i}+O_{i},$$

(2) $$S_{i}=S_{\mathrm{method}}+S_{\mathrm{experimental}}+S_{\mathrm{lightweight}}+S_{\mathrm{XAI}},\;0\leq S_{i}\leq 9.$$

Equation (3) expresses methodological information.

(3) $$M_{i}=\left\{\begin{array}{cc} 0.5, & n_{\mathrm{method}}\leq 4,\\ 1, & n_{\mathrm{method}}>4. \end{array}\right.$$

Equation (4) expresses used dataset count.

(4) $$D_{i}=\left\{\begin{array}{cc} 0.4, & \mathrm{single}\;\mathrm{dataset}\;,\\ 0.7, & \mathrm{two}\;\mathrm{datasets}\;,\\ 1, & \mathrm{multiple}\;\mathrm{datasets}\;(\mathrm{more}\;\mathrm{than}\;\mathrm{two}). \end{array}\right.$$

Equation (5) expresses hardware specifications.

(5) $$H_{i}=\left\{\begin{array}{cc} 1, & \mathrm{hardware}\;\mathrm{specifications}\;\mathrm{are}\;\mathrm{reported},\\ 0.5, & \mathrm{hardware}\;\mathrm{specifications}\;\mathrm{are}\;\mathrm{partially}\;\mathrm{reported},\\ 0, & \mathrm{otherwise}. \end{array}\right.$$

Equation (6) expresses software specifications.

(6) $$So_{i}=\left\{\begin{array}{cc} 1, & \mathrm{software}/\mathrm{environment}\;\mathrm{specifications}\;\mathrm{are}\;\mathrm{reported},\\ 0.5, & \mathrm{software}\;\mathrm{specifications}\;\mathrm{are}\;\mathrm{partially}\;\mathrm{reported},\\ 0, & \mathrm{otherwise}. \end{array}\right.$$

Equation (7) expresses peformance metrics.

(7) $$E_{i}=\left\{\begin{array}{cc} 0.5, & \mathrm{only}\;\mathrm{four}\;\mathrm{basic}\;\mathrm{metrics},\\ 0.75, & \mathrm{basic}\;\mathrm{metrics}\;\mathrm{and}\;\mathrm{at}\;\mathrm{least}\;\mathrm{one}\;(\mathrm{FPR},\;\mathrm{MCC}\;\mathrm{etc}.),\\ 1, & \mathrm{comprehensive}\;\mathrm{performance}\;\mathrm{evulation}. \end{array}\right.$$

Equation (8) expresses lightweight characteristics.

(8) $$L_{i}=\left\{\begin{array}{cc} 0, & \mathrm{no}\;\mathrm{clear}\;\mathrm{characteristics},\\ 0.5, & \mathrm{characteristics}\;\mathrm{without}\;\mathrm{quantitative}\;\mathrm{evidence},\\ 1, & \mathrm{characteristics}\;\mathrm{with}\;\mathrm{quantitative}\;\mathrm{evidence}. \end{array}\right.$$

Equation (9) expresses on-device testing applications.

(9) $$O_{i}=\left\{\begin{array}{cc} 1, & \mathrm{on}\text{-}\mathrm{device}\;\mathrm{testing}\;\mathrm{is}\;\mathrm{performed},\\ 0, & \mathrm{otherwise}. \end{array}\right.$$

Equation (10) expresses a count of the use of XAI methods.

(10) $$X_{i}=\left\{\begin{array}{cc} 0, & n_{\mathrm{XAI}}=0,\\ 0.5, & n_{\mathrm{XAI}}=1,\\ 1, & n_{\mathrm{XAI}}>1. \end{array}\right.$$

Equation (11) expresses XAI validations.

(11) $$V_{i}=\left\{\begin{array}{cc} 0, & \mathrm{no}\;\mathrm{validation}\;\mathrm{of}\;\mathrm{XAI}\;\mathrm{explanations},\\ 1, & \mathrm{expert}\;\mathrm{or}\;\mathrm{human}\;\mathrm{validation}\;\mathrm{is}\;\mathrm{performed}. \end{array}\right.$$

The methodological score ($S_{method}$) represents the extent to which a study evaluates its proposed approach across different methods and datasets. It is calculated as the sum of the methodological diversity ($M_{i}$) and dataset count ($D_{i}$) in Equation (12):

(12) $$S_{\mathrm{method}}=M_{i}+D_{i}.$$

The experimental score ($S_{experimental}$) assesses the completeness of the technical knowledge required to understand and reproduce the experimental setup and the results obtained. This score is calculated according to Equation (13), depending on whether the study reports the hardware ($H_{i}$) and software/environment specifications ($So_{i}$) used in the experiments and the experimental results ($E_{i}$).

(13) $$S_{\mathrm{experimental}}=H_{i}+So_{i}+E_{i}.$$

The lightweight score ($S_{lightweight}$) reflects the extent to which the proposed method is suitable for resource-constrained IoT environments. This score combines evidence supporting the lightweight design of the model ($L_{i}$) with evidence from on-device testing ($O_{i}$), as calculated in Equation (14).

(14) $$S_{\mathrm{lightweight}}=L_{i}+O_{i}.$$

The XAI score ($S_{XAI}$) assesses the extent to which the study under review considers explainability, and the quality of its supporting methodology. The variety of XAI techniques ($X_{i}$) is combined with the validation of the explanations generated ($V_{i}$), as in Equation (15).

(15) $$S_{\mathrm{XAI}}=X_{i}+V_{i}.$$

The Overall score ($S_{i}$) is obtained by combining the sub-evaluation scores, providing a structured representation of each study’s methodology, reproducibility, lightweight application, and explainability characteristics. The resulting score is intended to characterize the methodological and reporting features of the examined studies, rather than providing an absolute measure of their scientific validity.

## 4. Detailed Review of the Selected Studies

This section presents a detailed review and comparison of 48 of the recent studies from 2024 to the present, focusing on the IoT, IDSs, and XAI. Table 1 shows the bibliometric analysis data for these studies. All data in the table is taken from the JCR 2025 edition.

In [27], the study aimed to detect malicious bot activities in IoT environments and identify the bot family to which these activities belonged. The dataset was first subjected to preprocessing, the class imbalance problem was reduced, and feature selection was performed using XGBoost and correlation analysis. Among approximately 315 features, 16 important features were selected to improve the efficiency of the model. During the classification stage, an LSTM model whose hyperparameters were optimized using Bayesian optimization was employed. The model was reported to achieve a performance level of 99.99% in terms of accuracy, precision, recall, and F1-score. In addition, SHAP, an XAI method, was used to provide a clearer understanding of the features influencing the model’s decisions. In [28], a blockchain-supported, explainable, and DL-based intrusion detection framework was proposed to protect patient data and healthcare devices in IoMT environments. The proposed MA-DeepCRNN model learned spatial features using CNN, temporal dependencies using BiLSTM-RNN, and critical patterns through a multi-attention mechanism, while SHAP-based XAI analyses were used to make the model decisions more interpretable. It was reported that the model achieved an accuracy of 99.49% for binary classification, 99.12% for six-class classification, and 98.56% for nineteen-class classification in experiments conducted on the CICIoMT 2024 dataset. It was also reported that the blockchain structure had a block generation time of 10 s and provided a transaction throughput of approximately 182 TPS.

In [29], a lightweight and explainable intrusion detection model was developed for resource-constrained consumer IoT devices. Preprocessing, feature selection, and knowledge distillation (KD) were applied to the N-BaIoT and CIC-IDS 2023 datasets, and knowledge was transferred from a CNN-LSTM-based teacher model to a smaller student model. The student model achieved an accuracy of 99.87% on N-BaIoT and 98.71% on CIC-IDS 2023. Its size was reduced from 1.00 MB to 471.67 KB, while the inference time decreased from approximately 48 ms to 34 ms. SHAP was used to show that the student model retained the effects of the important features learned from the teacher model. In [30], an explainable deep learning ensemble model was proposed for detecting malware attacks in IoT networks. In the model, 1D-CNN, LSTM, and ANN were used as base learners, and their outputs were combined using a decision tree (DT) as the meta learner. It was reported that the model achieved an accuracy of 99.96% and an F1-score of 99.90% on the Aposemat IoT-23 dataset, while an accuracy of 99.57% and an F1-score of 99.50% were obtained on the AIoT malware detection dataset. LIME was used to explain the features that influenced the model decisions and to support the reliability of the proposed approach.

In [31], an explainable and lightweight IDS model was developed for IoT networks operating under resource constraints. In the approach, called DistillGuard, knowledge from a Transformer-based teacher model was transferred to a lighter student model using selective gradient-based knowledge distillation (SGKD). The study used the ToN-IoT, RT-IoT, and N-BaIoT datasets. It was reported that the student model achieved an accuracy of 96.43% on ToN-IoT, 98.81% on RT-IoT, and 99.98% on N-BaIoT in binary detection. In addition, gradient heatmaps, layer-wise contribution analysis, and gradient selection analysis were used to explain which layers contributed more to the detection process. In [32], the problems of improving accuracy, reducing false alarms caused by uncertain data, and increasing explainability in IoT-based IDS models were handled. The method employed a CNN-RNN-Transformer fusion structure known as CRT fusion and added a three-way clustering mechanism based on game-theoretic rough sets (GTRSs) to the final layer. In this way, network traffic was divided into attack, normal, and suspicious categories, while uncertain samples were left for further examination instead of being directly misclassified. It was reported that the model achieved an accuracy of 99.91% on CICIoT2023, 99.73% on TON-IoT, 99.96% on N-BaIoT, and 98.97% on X-IIoTID. SHAP and LIME were used to make the model decisions explainable.

In [33], the study aimed to detect DDoS attacks in IIoT networks using an explainable and lightweight model. In the system, SHAP and LIME were used together for feature selection, and the selected features were classified using a feedforward DNN model. In addition, low-contribution neurons were pruned using SHAP GradientExplainer to create the pruned HEAD model. It was demonstrated that the HEAD system achieved an accuracy of 98% on Edge-IIoTSet, 93% on HL-IoT, and 96% on ToN-IoT, while the inference time decreased after pruning. In [34], an explainable intrusion detection approach that considered both network layer and application layer data in IoMT environments was proposed. The ICU-IoT dataset was used, and raw packet and application layer features obtained from MQTT and CoAP traffic were processed. In the first stage, K-means was used together with PCA to visualize the separation of normal, malformed, MalariaDoS, and SlowITe traffic. Then, SHAP was used to select 13 features that were commonly influential across different scenarios from 52 features. NB, KNN, RF, AB, LR, and DT models were compared, and it was found that the KNN and RF models, in particular, achieved nearly perfect performance with the selected SHAP features, while their training and prediction times were reduced.

In [35], a deep learning-based IDS model was proposed to detect different types of attacks in IoMT and Internet of Health Things (IoHT) environments in an explainable manner. In the model, called XBiDeep, RNN, LSTM, GRU, BiLSTM, BiGRU, and hybrid architectures were tested, and the BiGRU-BiLSTM architecture, which produced the best results, was used. Multiclass classification was performed on the CICIoMT2024, IoMT-TrafficData, and ECU-IoHT datasets. It was reported that the model achieved an accuracy rate (AR) of 99.75% for six classes and 99.85% for nineteen classes on CICIoMT2024, an AR of 99.90% on IoMT-TrafficData, and an AR of 99.87% on ECU-IoHT. The model’s decision-making process was made more interpretable by explaining effective features at the global level using SHAP and at the attack-specific level using LIME. In [36], data integrity, model performance, and explainability issues in cyberthreat detection for smart healthcare systems were addressed together. A blockchain structure based on clique proof of authority (CPoA) was used to ensure the reliability of data obtained from multiple cloud providers. For attack detection, a deep learning model based on parallel stacked LSTM and a multihead self-attention (MhSA) mechanism was proposed and tested for binary classification on the ToN-IoT and IoT healthcare security datasets. It was shown that the model achieved an AR of 97.27% on the ToN-IoT dataset, and an AR of 89.62% on the IoT healthcare security dataset. SHAP was used to explain the features influencing the model’s decisions, with the aim of making the decision-support process more understandable for cybersecurity experts.

In [37], the study aimed to detect malicious traffic in IoT networks without centralized data collection and to make the model decisions explainable. A lightweight DNN model was trained within an FL framework using FedAvg, allowing clients to develop a shared model without exchanging their raw data. It was reported that the model achieved ARs of 99.3%, 99.5%, and 99.0% in binary classification on the Edge-IIoT, CIC-IoT2023, and TII-SSRC-23 datasets, respectively. For multiclass classification, the corresponding ARs were 97.2%, 98.0%, and 96.5%. SHAP and LIME were used to explain the traffic features that influenced the model’s attack predictions. In [38], an explainable deep learning-based IDS approach was proposed for multiclass attack detection in IoT networks. Label encoding, min–max normalization, and Pearson-correlation-based feature selection were applied to the NSL-KDD and UNSW-NB15 datasets. DNN, 1D-CNN, and 2D-CNN models were tested for classification. The highest AR was obtained using 2D-CNN, with a value of 99.40% on the NSL-KDDnew dataset and 81% on the UNSW-NBnew dataset. The DNN model was explained using LIME and SHAP, enabling the features affecting its decisions to be interpreted at both local and global levels. In [39], a lightweight, explainable, and highly accurate intrusion detection model was proposed for resource-constrained IoT devices. In the model, called DL-IID, DNN and BiLSTM were used together, while a wrapper-based genetic algorithm (GA) was applied for feature selection. The model size was reduced to 108.42 KB using post-training dynamic quantization. The model was tested on the RF Fingerprint, CICIDS2017, CICIoMT2024, and UNSW-NB15 datasets. It was reported that the model achieved an AR of 99.84%, a precision of 100%, a recall of 99.69%, and an F1-score of 99.84% on the RF Fingerprint dataset. In addition, LIME was used to explain the influence of features such as carrier frequency offset (CFO) and amplitude mismatch on the decision-making process.

In [40], a hybrid IDS model was proposed to make multiclass attack detection in IIoT networks more explainable and reliable. In the model, called XGRU-IDS, feature selection was performed using the extra trees classifier (ETC), sequential attack patterns were learned using gated recurrent units (GRUs), and model decisions were explained with SHAP. It was noted that experiments conducted on 34 classes in the CICIoT2023 dataset showed that the model achieved an AR of 97.56%, a precision of 97.41%, a recall of 97.56%, an F1-score of 97.42%, and a Cohen’s kappa value of 97.36%. SHAP made the multiclass detection results explainable at the class level. In [41], an explainable stacking ensemble IDS model was proposed to provide more reliable detection of different attack types in IoT networks. In the StaEn-IDS architecture, a deep IDS, CNN, and LSTM were used as base classifiers, and their outputs were combined using an RF meta classifier. The model was tested on the CIC-IoTIDS2022, NF-ToN-IoT, NF-BoT-IoT, NF-UNSW-NB15, NF-CSE-CICIDS2018, and NF-UQ-NIDS datasets. The best reported results were obtained on the NF-UNSW-NB15 dataset, with an AR of 99.49%, a precision of 99.47%, a recall of 99.49%, and an F1-score of 99.48%. The model was also converted to TensorFlow Lite and deployed on a Raspberry Pi 3. Its file size was reduced from 1.8 MB to 811 KB, and the average inference time was reported as 1.5 ms. In addition, LIME and SHAP were used to explain the network traffic features that influenced the model’s decisions.

In [42], the study aimed to detect DDoS attacks in network traffic using a lightweight, explainable, and real-time Transformer model. The model, called DistilXIDS, was developed based on DistilBERT. Flow-based features from the CIC-IDS2017, CIC-DDoS2019, and UNSW-NB15 datasets were converted into JSON-based textual representations and used for fine-tuning. Baseline, class-weighted, and SMOTE-based training strategies were compared. It was reported that the best AR results were obtained using the class-weighted strategy, with 99.74% on CIC-IDS2017 and 99.88% on CIC-DDoS2019, while the baseline strategy achieved the highest AR of 98.02% on UNSW-NB15. The SMOTE approach increased the F1-score of the DDoS class to 97.54%, particularly on the UNSW-NB15 dataset. SHAP and LIME were used to explain the model decisions, thereby improving reliability and interpretability for real-time IDS applications. In [43], the study aimed to develop a lightweight and explainable IDS for software-defined IoT environments. A feature selection method called SFOA-LASSO was proposed, in which SFOA was used to determine the appropriate α value for LASSO and reduce irrelevant features. Eight features were selected for SD-IoT devices and nine for SD-IoT servers. Binary and multiclass attack detection was then performed using DT, RF, XGBoost, and MLP models. It was reported that the RF model achieved an AR of 99.50% in binary detection and 99.49% in multiclass detection for SD-IoT devices. For SD-IoT servers, the RF model achieved an AR of 99.39% in binary detection and 99.38% in multiclass detection. SHAP summary and beeswarm plots were used to explain the features that influenced the model decisions.

In [44], the study aimed to perform device identification and attack detection in IoT networks using an explainable framework. The CICIoT2024-DIAD dataset was used, the 25 most important features were identified using RF, and the deep features extracted by CNN were passed to an XGBoost classifier. It was reported that the model achieved an AR of 99.92%, a precision of 99.62%, a recall of 99.13%, an F1-score of 99.97%, and a specificity of 99.27%. In addition, SHAP was used to explain the influence of features on the decision-making process. In [45], the study aimed to detect the C&C communications of IoT botnets at an early stage before an attack was launched. A semi-supervised anomaly detection approach trained only on benign traffic was used, and raw byte, packet, unidirectional flow, and bidirectional flow representations were compared. One-class SVM, isolation forest (IF), local outlier factor (LOF), elliptic envelope (EE), autoencoder (AE), and GAN models were tested. It was emphasized that the best result was obtained using packet-level OSVM, which achieved a recall of 99.99% and a false-positive rate (FPR) of 1.53% for all malicious traffic. For early-stage C&C detection, the model achieved a detection rate (DR) of 99.51% and an FPR of 1.09%. Feature selection, a surrogate model, permutation importance, and LIME were used to improve explainability.

In [46], an IDS framework was proposed to jointly address class imbalance, irrelevant features, hyperparameter tuning, and limited labeled data in IoT environments. In the DOAL-IDS framework, low-variance features were first removed using the variance threshold (VT) method, the data were balanced with ProWRAS, and the capsule network (CapsNet) model was optimized using the reptile search algorithm (RSA). A margin-based active learning (MBAL) approach was then integrated to develop MARCO-Net, with the aim of reducing labeling costs by selecting the most uncertain samples for annotation. It was reported that the model achieved an AR of 87% and an F1-score of 87% on the ToN_IoT dataset, while MARCO-Net reached an AR of 90% and an F1-score of 90%. SHAP and LIME were used to make the model decisions explainable. In [47], a lightweight and explainable network IDS was proposed for resource-constrained environments such as edge gateways. In the FCAST-RF approach, PCA was used for dimensionality reduction, contextual traffic information was added through a sliding window, and the RF model was optimized using a multithreshold evaluation (MTE) strategy. It was revealed that the model achieved an accuracy of 98.60%, a macro F1-score of 97%, a macro precision of 98.30%, and a macro recall of 95.70% on the CICIDS2017 dataset. The effects of the features on the decision-making process were evaluated using SHAP-based explanations.

In [48], the study developed a generalizable and explainable IDS model for different IoT domains. In the method, called X-FuseRLSTM, two different feature groups were extracted using a deep encoder and a sparse Transformer. Then, these features were combined, reduced using PCA, and classified with a residual LSTM model. It was emphasized that the model achieved an accuracy of 99.40% on TON_IoT Network, 99.72% on NSL-KDD, 97.66% on the 19-class CICIoMT 2024 problem, and 98.05% on the six-class problem. SHAP and LIME were used to make the model decisions more interpretable. In [49], the study aimed to reliably classify phishing websites in IoT-enabled cloud-based CPS. In the method, called XAIAOA-WPC, data cleaning, text preprocessing, and standardization were applied. Important features were identified using Harris Hawks Optimization (HHO)-based feature selection, and the multihead attention LSTM model was optimized using the Aquila Optimization algorithm (AOA). It was reported that the model achieved an AR of 98.92% with an 80–20 train–test split and a maximum AR of 99.29% with a 70–30 split on the phishing URL dataset. LIME was used to explain the model’s decisions for phishing and legitimate URLs. In [50], a lightweight intrusion detection model was developed for use in conventional networks, IoMT, and SDN environments. In the lightweight deep CNN model, called LWIDS-DCNN, a 1D-CNN-based architecture was used together with feature selection, one-hot encoding, and standardization. It was noted that the model achieved an AR of 99.93% on CICIDS2017, 99.70% on CICIoMT2024, and 99.97% on InSDN.

In [51], a privacy-preserving and explainable anomaly detection model was developed for CPS-enabled IoT networks. In the framework, called CPS-IoT-PPDNN, feature dimensionality was reduced using an LSTM-AE, a DNN model was trained, and the model decisions were explained using deep SHAP. It was reported that the model achieved 100% AR, precision, recall, and F1-score in binary classification on the Edge-IIoTset and X-IIoTID datasets. In multiclass scenarios, it achieved approximately 99.98% accuracy on Edge-IIoTset and 99.99% on X-IIoTID. In [52], an explainable and adaptive IDS model was developed to reduce dependence on labeled data in IoT networks. In the model, called EADL-IDS, unsupervised binary anomaly detection was performed using a denoising convolutional autoencoder (DCAE), attack data were clustered using K-means, and attack types were explained through an LLM-based retrieval augmented generation (RAG) approach. It was found that the model achieved over 95% performance in binary classification and over 85% in multiclass classification on the NSL-KDD, CIC-IoT-2023, and ACI-IoT-2023 datasets.

In [53], an explainable and optimized TabNet-based IDS model was proposed for anomaly-based attack detection in IoT environments. The hyperparameters of the TabNet Transformer model were optimized using Google OSS Vizier, and the model was tested for both binary and multiclass classification on the NSL-KDD, CICIoT2023, and RT_IoT2022 datasets. It was reported that the optimized TabNet model achieved ARs of 99.90% on NSL-KDD, 99.62% on CICIoT2023, and 99.16% on RT_IoT2022 in binary classification. For multiclass classification, the corresponding AR values were 98.29%, 98.43%, and 98.10%, respectively. SHAP was used to explain the features that influenced the model’s decisions. In [54], an explainable IDS model that could operate in real time on resource-constrained IoT devices and remain robust to class imbalance was developed. The DS2OS and ToN_IoT datasets were used. Conditional tabular GAN (CTGAN) was applied to generate synthetic data and balance minority classes, while K-means was used for unsupervised feature generation. Several models, including LightGBM, XGBoost, linear discriminant analysis (LDA), DT, KNN, AdaBoost, LSTM, quadratic discriminant analysis (QDA), and TabNet, were compared. It was emphasized that the models generally achieved accuracies ranging from 97.20% to 100% on ToN_IoT and above 99.41% on DS2OS. Based on the results obtained on Jetson Nano, the best performing models were considered suitable for edge environments, with inference times ranging from 0.0097 to 0.5686 s and model sizes ranging from 2.73 to 1510 kB. SHAP and LIME were also used to explain the model decisions.

In [55], an explainable network IDS approach supported by incremental learning was investigated to enable adaptation across different IoT network environments. The IoT-NID, TON_IoT, and Edge-IIoT datasets were used, and the behavior of a 2D-CNN-based model trained on a source network was evaluated on a target network under class incremental learning (CIL) and domain incremental learning (DIL) scenarios. It was reported that the F1-score remained above 79% in binary and multiclass evaluations conducted within the same dataset. However, when the model was transferred to a different dataset, its performance decreased substantially to approximately 33%. Incremental learning approaches increased the F1-score to above 71%. Fine-tuning with memory (FT-Mem) was found to be more effective for complex source networks, whereas bias correction (BiC) performed better for simpler source networks. In addition, SHAP analysis showed that traffic-related features played a decisive role in the classification process. In [56], the study aimed to detect IoT botnet attacks within a federated learning framework while preserving data privacy and making the model decisions explainable. In the FedXAI framework, an LSTM model was trained using FedAvg in a horizontal FL environment. It was noted that the model achieved an AR of 99.90% and an F1-score of 99.99% in binary classification on the N-BaIoT dataset. Regarding botnet detection, it achieved an accuracy of 99.28% and an F1-score of 99.54%, while for attack detection, it achieved an AR of 94.89% and an F1-score of 94.50%. The study combined a privacy-preserving FL structure with XAI-based explanations.

In [57], an intrusion detection framework was proposed to jointly handle class imbalance, the need for labeled data, hyperparameter tuning, and explainability in resource-constrained IoST environments. In the study, data imbalance was reduced using ADASYN, while a logistic regression (LR) model was enhanced through entropy-based active learning, uncertainty-based active learning, and sandpiper optimization. It was reported that LRALE, LRALU, and LRSO achieved ARs of 92%, 91%, and 84%, respectively, on the IoSTs dataset. On the CIC-IDS-DS dataset, the corresponding AR values were 98%, 97%, and 97%. LIME and SHAP were used to explain the features influencing the model decisions, resulting in a more interpretable IDS approach. In [58], the study aimed to detect cyberattacks in IoT networks with high accuracy while also making the model decisions explainable. The NF-BoT-IoT-V2 dataset was used, and ANN and 1D-CNN methods were employed for model training. It was noted that both models achieved an AR of approximately 98%, while the macro F1-scores were 76% for ANN and 77% for CNN. In addition, SHAP and LIME were used to identify the features that had the greatest influence on model decisions. In [59], a lightweight IDS capable of operating on resource-constrained IoT devices was developed. Following data preprocessing, K-means-based sampling was applied, the ten most important features were selected from three datasets using the mFCBF method, and class imbalance was reduced using SVM-SMOTE. LightGBM and XGBoost were used in the classification. It was emphasized that the AR achieved an accuracy of 99.34% on CICIDS2017, 99.62% on NSL-KDD, and 99.75% on BoT-IoT. In addition, an average CPU consumption of 2.03% and memory usage of approximately 1.2% were noted.

In [60], the study aimed to detect both high-volume and low-volume DDoS attacks in consumer IoT networks in an explainable manner and in a form suitable for edge devices. In the ADEPT system, CNN and LSTM models were combined through an attention-based ensemble architecture, and the model was optimized using differential evolution-based pruning and min–max quantization. The ToN-IoT and CICIoT-2023 datasets were combined into a dataset called TTC. It was reported that the method achieved an AR of 98% on ToN-IoT, 93% on CICIoT-2023, and 94% on TTC. On a Raspberry Pi 4, the system achieved a latency of 0.57 s, a throughput of 1775 samples per second, and memory usage of 746 MB. SHAP and risk assessment plots were used to make the model decisions more understandable. In [61], an explainable ensemble IDS framework was proposed to classify different types of cyberattacks with low computational cost. Important features were first selected using mutual information (MI), after which PCA was applied to reduce the feature space to five components. A hard voting ensemble model based on DT, XGBoost, and LightGBM was then used for classification. It was demonstrated that the model achieved an AR of 99.92% on the MSCAD-2022 dataset and 99.97% on the CICIDS-2017 dataset. SHAP was used to explain how the individual components influenced the model decisions. In [62], the study aimed to detect cyberthreats in IoT environments through a secure, explainable, and optimized framework. In the TTOCDM-XAIRNN method, blockchain was used for secure data transmission, LSN was applied for preprocessing, POA was used for feature selection, and the A-LSTM-BiGRU model employed for cyberthreat detection was optimized using EOA. It was reported that the model achieved an AR of 98.34% on the NSL-KDD dataset and 98.87% on the CICIDS-2017 dataset. SHAP was used to explain which features influenced the model decisions.

In [63], a real-time, lightweight, and adaptive IDS architecture was developed for IoMT environments. Class imbalance was reduced using SMOTE, ten important features were selected using RF and permutation importance, and C4.5, XGBoost, RF, voting ensemble, and neural network models were compared. The C4.5 model was selected as the most suitable low-latency structure and was later integrated with DQN-based RL to develop the hybrid C4.5-DQN IDS. It was stated that this hybrid model achieved an AR of 99.20% in the binary classification scenario on the CICIoMT2024 dataset. In [64], the study aimed to detect cyberattacks in IoMT-based healthcare systems using a blockchain-supported, secure, and explainable ADS framework. The CICIoMT-2024 dataset was used, and label encoding, mutual-information-based feature selection, min–max normalization, and correlation-based feature reduction were applied. An XGBoost model optimized using Optuna was employed for classification. In addition, a blockchain-based secure recording structure was created using Nik512 hashing, ZKP verification, trust-weighted consensus, and off-chain storage. It was reported that the model achieved 99.90% AR, 99.78% precision, 99.89% recall, an F1-score of 98%, and 99.98% specificity in the 2-, 6-, and 19-class scenarios. SHAP and LIME were used to make the model decisions explainable. In [65], a SaaS-based and explainable IDS capable of operating in edge environments was developed for resource-constrained IoMT devices. After data preprocessing, PSO was used for feature selection, and LR, SVM, DT, RF, KNN, and ELM models were compared. It was stated that the PSO-DT model achieved the best results on the WUSTL-EHMS-2020 dataset, with 96.56% AR, 97.78% precision, 98.26% recall, and an F1-score of 98.02%. SHAP was used to show which features influenced the model decisions.

In [66], a five-stage framework called FORT-IDS was proposed, combining feature alignment, SMOTE, adversarial training, SHAP and LIME explanations, GNN, federated learning, continual replay, pruning, and quantization. In tests conducted on the UNSW-NB15 and DDoS Botnet IoT datasets, the F1-scores for LR, RF, and MLP were reported as 80%, 91%, and 95%, respectively. In cross-dataset tests, the F1-score decreased to 39% when the model was trained on the DDoS dataset and tested on UNSW-NB15, whereas an F1-score of 99% was obtained in the reverse setting. In [67], an explainable IDS framework was proposed to jointly address high-dimensional data, class imbalance, and model explainability in IoT networks. In the OptiXID framework, feature selection was performed using ASO, class imbalance was reduced using safe-level SMOTE, and XGBoost, DT, RF, and LightGBM models were compared. It was reported that the XGBoost model achieved 98.4% AR, 98.6% precision, 98.4% recall, and an F1-score of 98.6% on the NF-UNSW-NB15 dataset. On the CIC-IoT-2023 dataset, it achieved 99.48% AR, 99.47% precision, 99.48% recall, and an F1-score of 99.47%. SHAP was used to make the IDS results more reliable and interpretable. In [68], a lightweight and explainable IDS model was developed to reduce dependence on manual feature selection in IoT networks. The SA-DNN model was enhanced with a learnable feature gating (LFG) mechanism. CFS-based feature selection was used for BoT-IoT, RF-based feature selection was applied to N-BaIoT, and sampling methods, SHAP, and LIME were employed. It was stated that the model achieved an AR of 99.3% on BoT-IoT, 99.6% on N-BaIoT, and 97.9% on UNSW-NB15. SHAP and LIME were used to make the model decisions more explainable for the IoT datasets.

In [69], a framework was developed to jointly handle the requirements of accuracy, explainability, and deployability in edge environments for IDS models. In the HED-ID method, temporal traffic patterns were learned using S-BiGRU and an attention mechanism, while GWO was used for feature selection and hyperparameter tuning, and SHAP was employed to explain the model decisions. It was reported that, after GWO optimization, the AR reached 92.1% on UNSW-NB15, 95.6% on CICIDS-2017, 93.1% on ToN-IoT, 94.8% on CIC-IDS2018, and 93.5% on the full TON_IoT dataset. In addition, ARs of 95.6% in a cloud environment and 93.8% in an edge environment were stated, together with a latency of 18–22 ms and memory usage of 92–115 MB. In [70], the study aimed to detect anomalies and attacks targeting sensor communication in IoT-enabled autonomous transportation systems in a real-time and explainable manner. In the EAI-IDS model, temporal sensor patterns were analyzed using LSTM, feature-level anomalies were examined using RF, and SHAP was used to explain the variables influencing the model decisions. It was emphasized that the model achieved 94.2% AR, 92.4% precision, 93.5% recall, an F1-score of 92.9%, a latency of 15.2 ms, energy consumption of 2.1 J, and a packet delivery ratio of 98.5%. In [71], the study aimed to detect network attacks and identify attack types in IoT-based metaverse environments. A two-layer architecture was developed, in which CNN was used for feature extraction in the first layer, while CatBoost and AdaBoost classifiers were employed in the second layer. The models were optimized using metaheuristic algorithms, particularly DAPSO. It was noted that, on the CICIoT2023 dataset, ARs for binary classification were 99.32% for CatBoost and 99.28% for AdaBoost. For multiclass classification, the corresponding ARs were 87.68% and 87.49%. In addition, SHAP was used to examine the features that influenced the model decisions.

In [72], the study aimed to reduce the vulnerability of machine learning-based IDS models, particularly against black-box adversarial attacks. RF was used for attack detection, while a SHAP-based credibility assessment module (CAM) was employed to evaluate the reliability of the prediction. When the credibility level was low, a second line of defense was activated using window-based classifiers and a GPT-2-based payload analyzer. It was demonstrated that the framework achieved an AR and F1-score of 99.05% on the CSE-CIC IDS 2018 dataset, while it achieved an AR of 98.15% and an F1-score of 98.14% on the CIC-IoT 23 dataset. In addition, the evasion rates under HopSkipJump and ZOO attacks were limited to 1.9% and 6.2%, respectively, on CSE-CIC IDS 2018, and to 6.4% and 5.5% on CIC-IoT 23. In [73], a multilayer IDS framework called DeepGuard was proposed to address attack detection, attack classification, and alert generation in heterogeneous IoT and IIoT environments. The ten most important features were selected using RF, class imbalance was reduced using SMOTE, and an MLP model with two hidden layers was used for attack classification. It was reported that the model achieved an AR of 99.99% on the WUSTL-IIoT-2021 and X-IIoTID-2022 datasets in a PC i7 environment. F1-scores of 99.90% for X-IIoTID and 99.95% for WUSTL were also reported. In [74], the study aimed not only to detect attacks in IIoT and CPS environments but also to classify them in an explainable manner according to their impact levels and behavioral characteristics. Hidden traffic patterns were examined using K-means and DBSCAN. Subsequently, 33 attack types were grouped into seven broader categories and classified using RF, XGBoost, and MLP models. It was stated that the overall AR on the IoT intrusion detection dataset was 99.48% for RF, 99.51% for XGBoost, and 99.15% for MLP. However, recall values were low for minority classes, particularly injection attacks, backdoors, and exploits. The study presented an interpretable framework for IIoT and CPS security.

The 48 studies reviewed implement IDSs in IoT systems and make these processes explainable. Detailed analyses and comparisons of these studies are presented in Table 2.

In this paper, the review of the selected studies is not limited to summarizing their content. Due to substantial heterogeneity across the included studies in terms of datasets, attack types and numbers of classes, training–test splits, models, preprocessing methods, and experimental settings, a meta-analysis or pooled effect estimate was not considered appropriate. Performance metrics, including accuracy, precision, recall, F1-score, and the AUC, were therefore reported descriptively as presented in the original studies. Furthermore, beyond these metrics, the strengths and weaknesses of the studies examined are indicated, a quality score is calculated according to our own defined quality score assessment, and the studies are analyzed accordingly. In addition, a comprehensive assessment is carried out by considering whether the studies adopt lightweight model approaches and which XAI methods they use. These evaluations are shown in Table 3. To ensure consistency in data presentation in all tables, performance values originally reported as proportions were converted into percentages, and all numerical values were rounded to two decimal places. Information that was not reported or could not be identified in the original studies was indicated as “not specified” or marked with “✗” in the corresponding tables.

To ensure a consistent interpretation of lightweight characteristics across the reviewed studies, the studies are classified as ✓, ◐, or ✗ in the Lightweight column of Table 3, based on the evidence provided in the original articles. Studies are classified as ✓ when lightweight or resource-efficient operation is explicitly addressed and supported by relevant computational or resource-related evaluations. Studies are classified as ◐ when they include mechanisms related to computational efficiency, such as feature selection, pruning, dimensionality reduction, or model simplification, but do not provide sufficient evidence to fully demonstrate lightweight operation. Studies are classified as ✗ when no clear lightweight characteristics, resource efficiency assessments, or related evaluations are identified. Importantly, this classification reflects the lightweight characteristics of the proposed IDS design and does not indicate that the computational overhead of the associated XAI method was evaluated separately.

Explanation validation is assessed separately from the use of XAI techniques. Studies are classified as ✓ when the explanations generated by the XAI methods are explicitly evaluated using a specific validation procedure, such as fidelity, faithfulness, stability, robustness, consistency, sensitivity, quantitative explanation quality measures, comparison between different explanation methods, or human or expert evaluation. Studies are classified as ✗ when XAI methods are applied but the analysis is limited to feature importance rankings, SHAP or LIME plots, local explanations, or qualitative interpretation of the explanations. Predictive performance metrics, cross-validation results, model robustness, and general statements describing a model as explainable are not considered evidence of explanation validation for they directly evaluate the generated explanations.

## 5. Results and Discussion

Recent studies examining IDSs developed for IoT-based environments reveal that research focuses not only on classification performance but also on concepts such as model explainability, real-time operability, resource efficiency, and data privacy. In particular, the integration of XAI techniques into IDS models reduces the “black box” nature of deep learning models, enabling security analysts to interpret the decisions produced by the model. In this context, SHAP and LIME stand out as the most commonly used explainability methods, while recently, attention mechanisms and LLM-based explanation methods have begun to be used.

A large portion of the studies in the literature utilize traditional ML methods, CNN, LSTM, GRU, BiLSTM-based deep learning frameworks, or hybrid combinations thereof. While CNN-based frameworks have proven successful in network traffic studies, LSTM and GRU-based models stand out in time-series studies. In many studies, the use of ensemble approaches instead of a single model has enabled the development of more stable and higher-accuracy IDSs by combining the strengths of different models.

With the proliferation of resource-constrained IoT devices, developing lightweight IDS models has become a focal point for researchers. The reviewed studies addressed lightweight design through different strategies, including feature selection, dimensionality reduction, model pruning, knowledge distillation, quantization, model simplification, and edge-oriented deployment. In some studies, resource efficiency was further supported by measurements such as inference time, latency, memory consumption, model size, or execution on edge devices. However, the reviewed studies generally do not evaluate whether these optimizations affect explanation quality. Thus, improvements in computational efficiency do not necessarily indicate that explanation fidelity, stability, or consistency is preserved. This situation is also reflected in the explanation validation results, as only 10 of the 48 reviewed studies validated the generated explanations.

**Figure 4:**
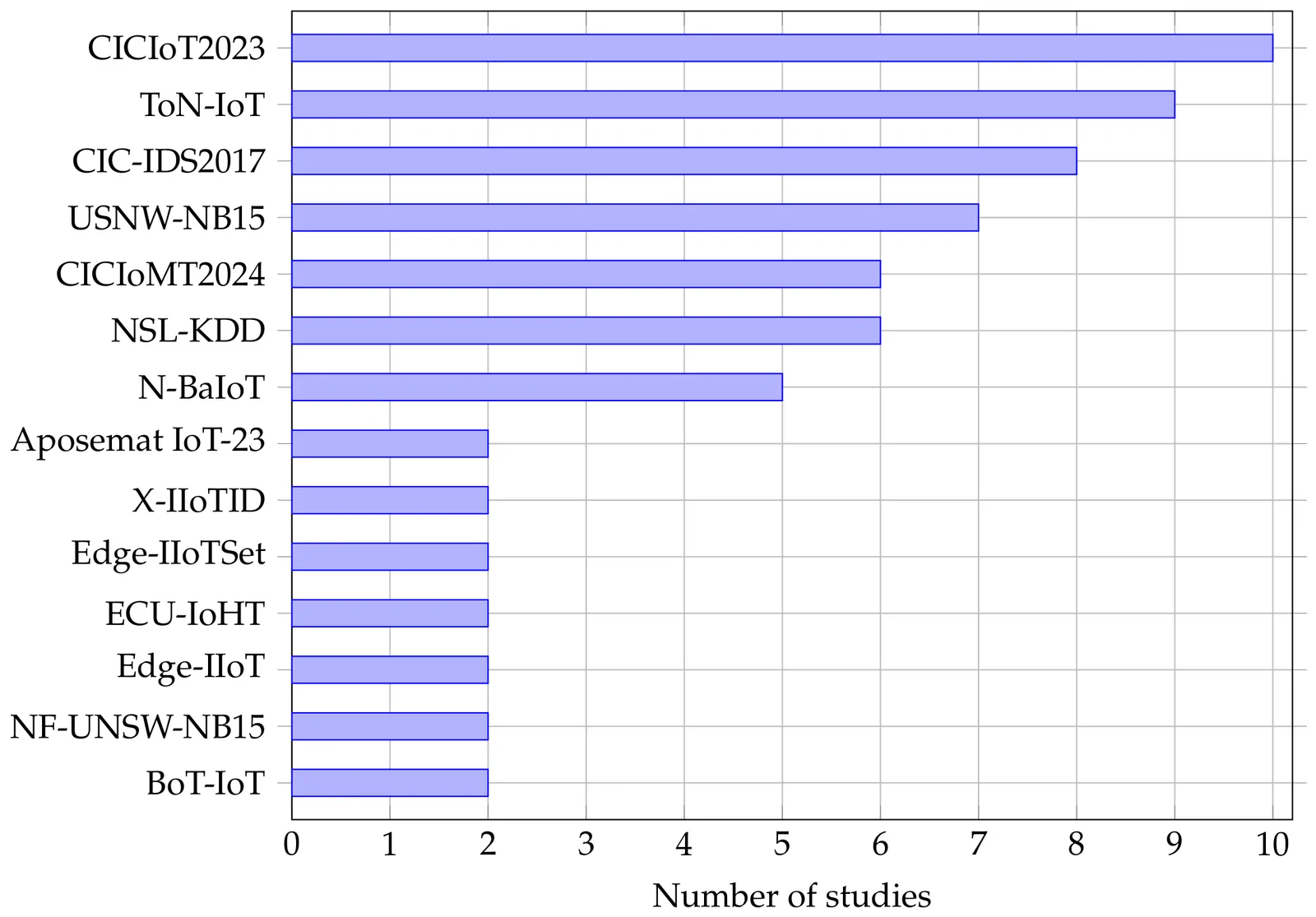
Frequency of datasets used in the reviewed studies.

**Figure 5:**
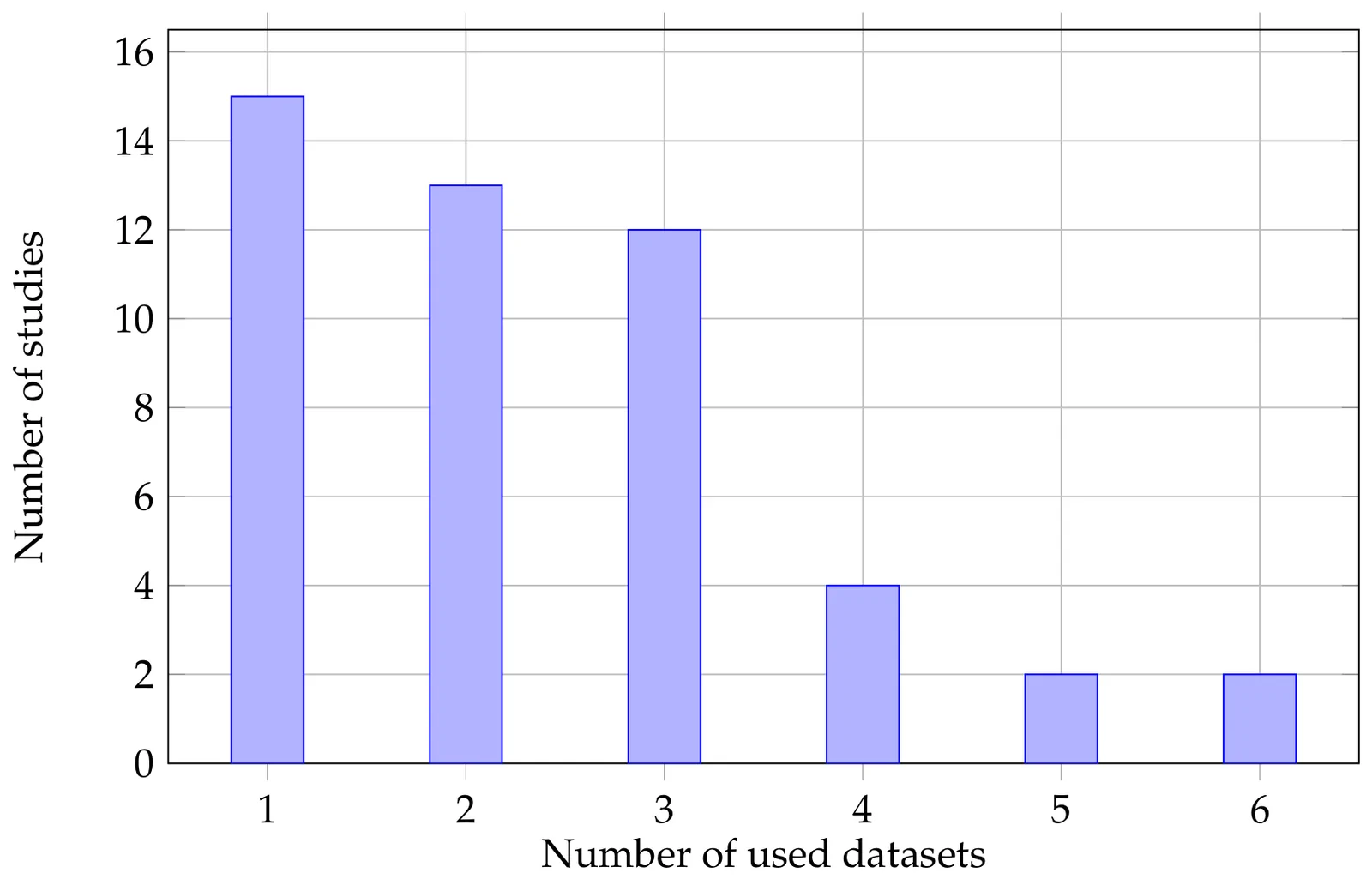
Distribution of the number of occurrences among 48 studies.

Using SMOTE, ADASYN, CTGAN, and GAN-based data generation methods, the goal is to more successfully learn attack classes with a small number of examples. Figure 4 shows the number of times datasets were used multiple times overall in the studies. CICIoT 2023 and ToN-IoT datasets stand out as the most commonly used datasets. Figure 5 shows the number of studies examined according to the number of datasets used. Many studies obtained results with a single dataset, while some studies used five or six different datasets. However, most of the existing studies were evaluated on balanced laboratory datasets, and the development of IDS models for the constantly changing network traffic structure and new attack types in a real network environment remains a significant and current research problem.

In recent years, there has been a rise in blockchain- and federative learning-based IDS approaches aimed at protecting data privacy. In IoMT studies, particularly those developed for healthcare systems, blockchain technology is used for secure data sharing, while federative learning approaches enable collaborative model training without centralized data collection. However, since a significant portion of these studies do not address metrics such as model synchronization, communication costs, and performance losses in detail, there is a need for optimized approaches in this field. Ref. [75] reveals that adding privacy and explainability layers in edge environments increases delays by 70–80%, significantly contributing to the debate on the resource costs and delays associated with these features.

**Figure 6:**
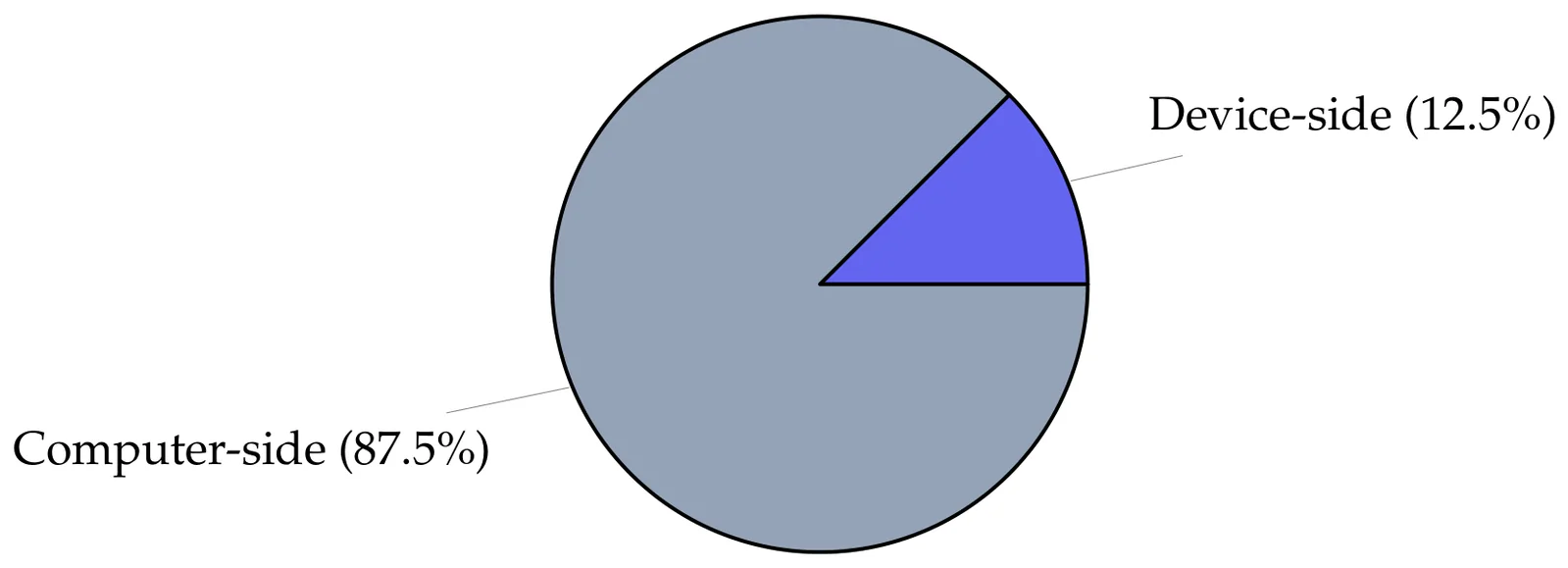
Distribution of test information by environment.

**Figure 7:**
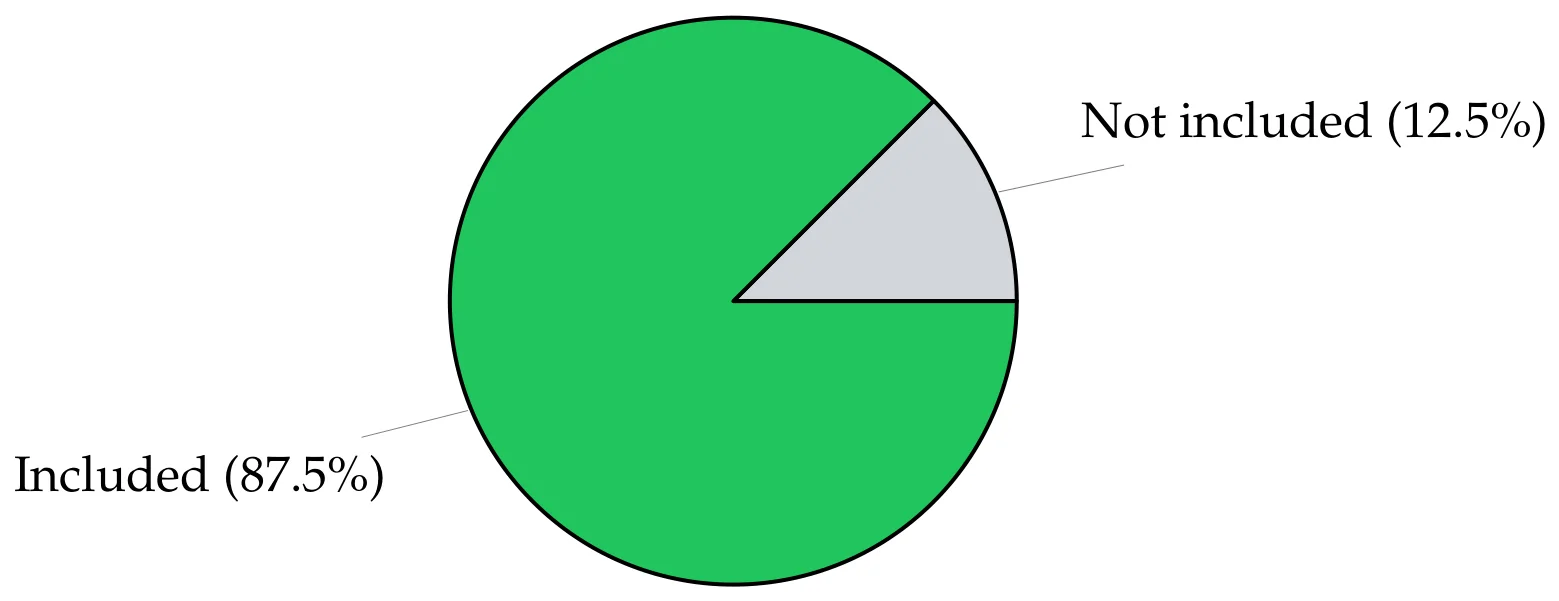
Comparison of studies reporting software and hardware specifications.

In this paper, to ensure consistent classification, XAI adoption is determined based on whether an explainability method is actually implemented in each study rather than merely mentioned. Therefore, some studies retrieved through the search strategy are classified as not implementing XAI after full-text examination. The experimental settings of the reviewed studies were classified as device-side or computer-side based on the environment in which the proposed IDS is evaluated. Studies involving physical deployment or testing on resource-constrained edge or IoT devices are classified as device-side, whereas studies evaluated in computer-based experimental or simulation environments without physical edge device deployment are classified as computer-side. Figure 6 shows the distribution of whether tests were performed or not on any device in all studies. It is noteworthy that the majority of studies were simulated in a computer environment rather than being tested on any device. In addition, Figure 7 shows whether information about the hardware and software environments in which the tests were performed was provided in the studies examined. As a result of the examination, it can be seen that the studies predominantly shared this information.

Most studies show that model decisions are explained using SHAP and LIME approaches. However, explainability is generally limited to showing the order of importance of features. The contribution of explanations to the decision-making processes of security analysts, the consistency of different XAI methods, and the impact of explanations on attack prevention processes are evaluated in only a few studies. Recently developed LLM-supported explanation systems and RAG-based approaches are important steps towards addressing this deficiency, but more research is needed in this area.

Given that the primary focus of this systematic review is on explainability and lightweight design in IoT-based intrusion detection systems, the sensitivity analysis is specifically focused on these two dimensions. Rather than examining pooled performance estimates, which was considered inappropriate due to the substantial methodological and experimental heterogeneity among the reviewed studies, the analysis assesses the state of the main synthesis findings to alternative classification criteria. In particular, the classification of studies as "lightweight" is examined under two different criteria. Of the 48 reviewed studies, 35.42% are classified as lightweight, 37.50% as partially lightweight, and 27.08% as non-lightweight. When partially lightweight studies are included in the lightweight category, 72.92% of studies meet the broader criterion. In contrast, under a stricter criterion considering only studies explicitly classified as lightweight, this proportion decreases to 35.42%. This substantial difference indicates that conclusions regarding the prevalence of lightweight IDS approaches are sensitive to the classification criterion, and should therefore be interpreted with caution.

The state of the findings concerning XAI is also examined based on the distribution of explainability methods. An XAI approach was adopted in 44 of the 48 studies. However, the distribution of XAI methods is highly concentrated. Of the 44 studies incorporating XAI, 86.36% employ SHAP, either alone or in combination with LIME. Specifically, 22 studies employ SHAP without LIME, 16 combine SHAP and LIME, four employ LIME without SHAP, and two use other XAI approaches. Thus, while the conclusion that XAI is widely incorporated into recent IoT-based IDS studies remains robust, the evidence base is generally focused on SHAP-based explanations.

An important point emerging from this review is that the lightweight characteristics of an IDS and the computational overhead introduced by its explainability component should be evaluated separately. In the reviewed studies, lightweight characteristics were identified based on the reported evidence, including resource efficiency evaluations and strategies aimed at reducing computational requirements, such as feature selection, pruning, dimensionality reduction, and model simplification. However, the computational cost specifically associated with XAI methods was not consistently evaluated across the literature. Although some studies reported measures such as execution time, latency, or memory consumption, many did not separately assess the additional cost of generating explanations. This is particularly important for methods such as SHAP and LIME, which may require additional computational resources. Therefore, classifying an IDS as lightweight does not necessarily mean that the entire system, including its XAI component, is suitable for resource-constrained edge devices. Future studies should also evaluate the additional latency, memory consumption, and computational overhead introduced by XAI under realistic edge or IoT deployment conditions.

The explanation validation column in Table 3 reveals a significant gap between the adoption of XAI and the validation of the explanations it produces. Only 20.8% of the 48 studies examined validated the generated explanations, while 79.2% did not undergo separate explanation validation. Most studies address explainability through the presentation and interpretation of feature significance or local and global explanations. The quality and reliability of these explanations are generally detailed in terms of aspects such as fidelity, stability, robustness, or consistency. Human or expert assessments remain limited. These findings indicate that the use of XAI in IoT-based IDS research is progressing faster than the systematic validation of the explanations it produces. Therefore, the application of an XAI method alone should not be considered sufficient evidence that an IDS provides reliable or practically useful explanations. Future studies should support the use of XAI techniques with validation of explanation quality using quantitative measurements and, where appropriate, human or expert evaluation. In [76], it is reported that human validation is not common in security-focused XAI research on this topic. Furthermore, it is shown that different explanation methods can produce inconsistent explanations for the same predictions, and that applying an XAI method alone does not guarantee the reliability or practical usefulness of the resulting explanations.

Of the 17 studies classified as lightweight, only four explicitly assess the computational cost associated with XAI components, and three of these present results demonstrating that lightweight operation can be sustained even after considering the XAI cost. This indicates that the computational overhead of explainable IDS design is not sufficiently examined, even in studies that provide strong evidence for lightweight IDS design. However, in many cases, it is noted that computationally demanding XAI operations are assigned to relatively high-resource servers, cloud platforms, or central aggregation nodes instead of resource-constrained IoT or edge devices. Therefore, the absence or high level of XAI-specific overhead metrics may not necessarily mean that lightweight operation of the edge IDS is compromised. However, this also does not provide direct evidence that the entire IDS-XAI pipeline is lightweight. Accordingly, the lightweight classification used in this review reflects the computational and resource efficiency characteristics of the IDS model and the overall deployment architecture. The computational overhead specifically introduced by XAI should therefore be considered as a separate dimension requiring more systematic evaluation in future studies.

**Figure 8:**
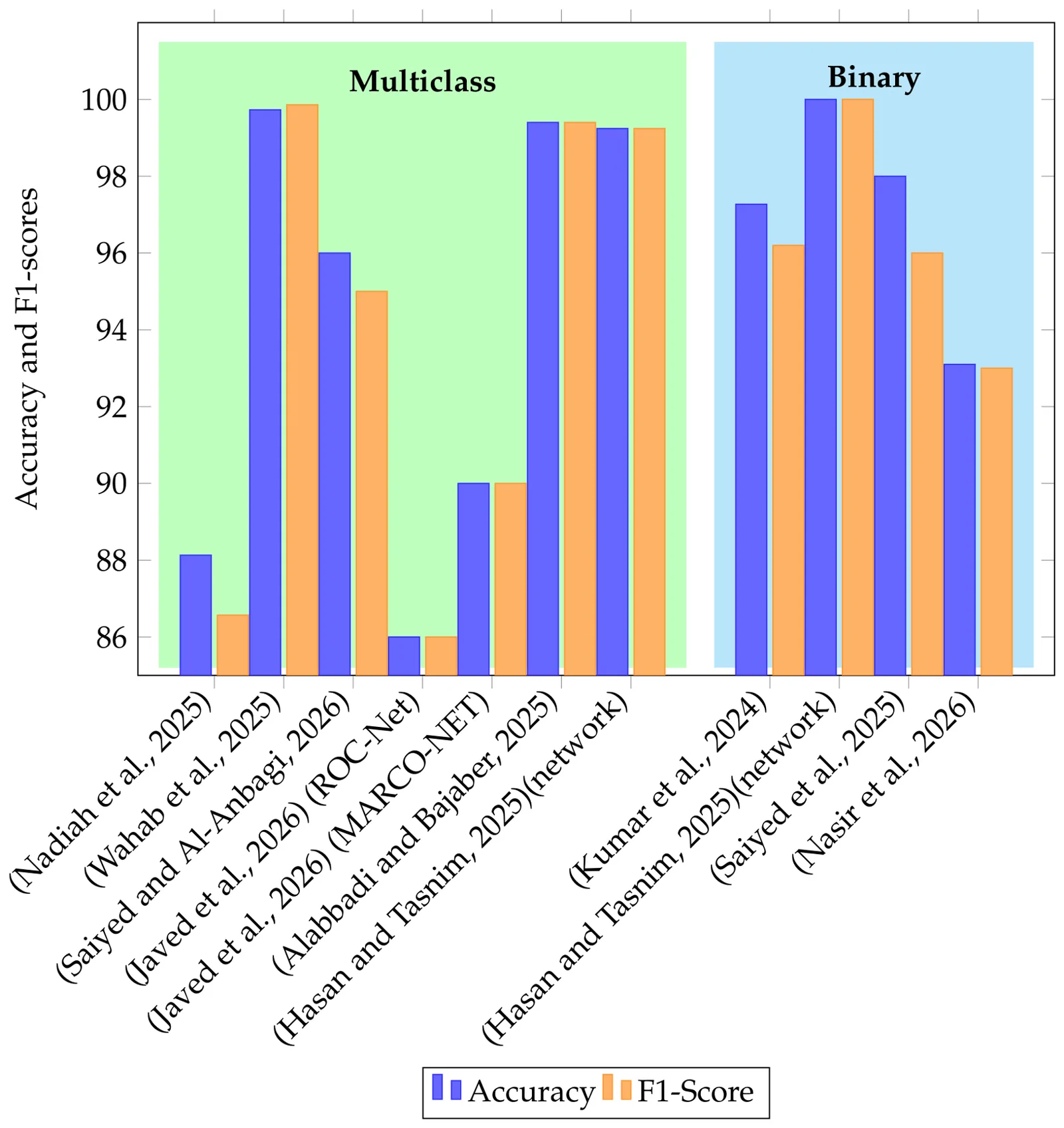
Comparison performance metrics of studies ([31,32,33,36,46,48,54,60,69]) using the ToN-IoT dataset.

**Figure 9:**
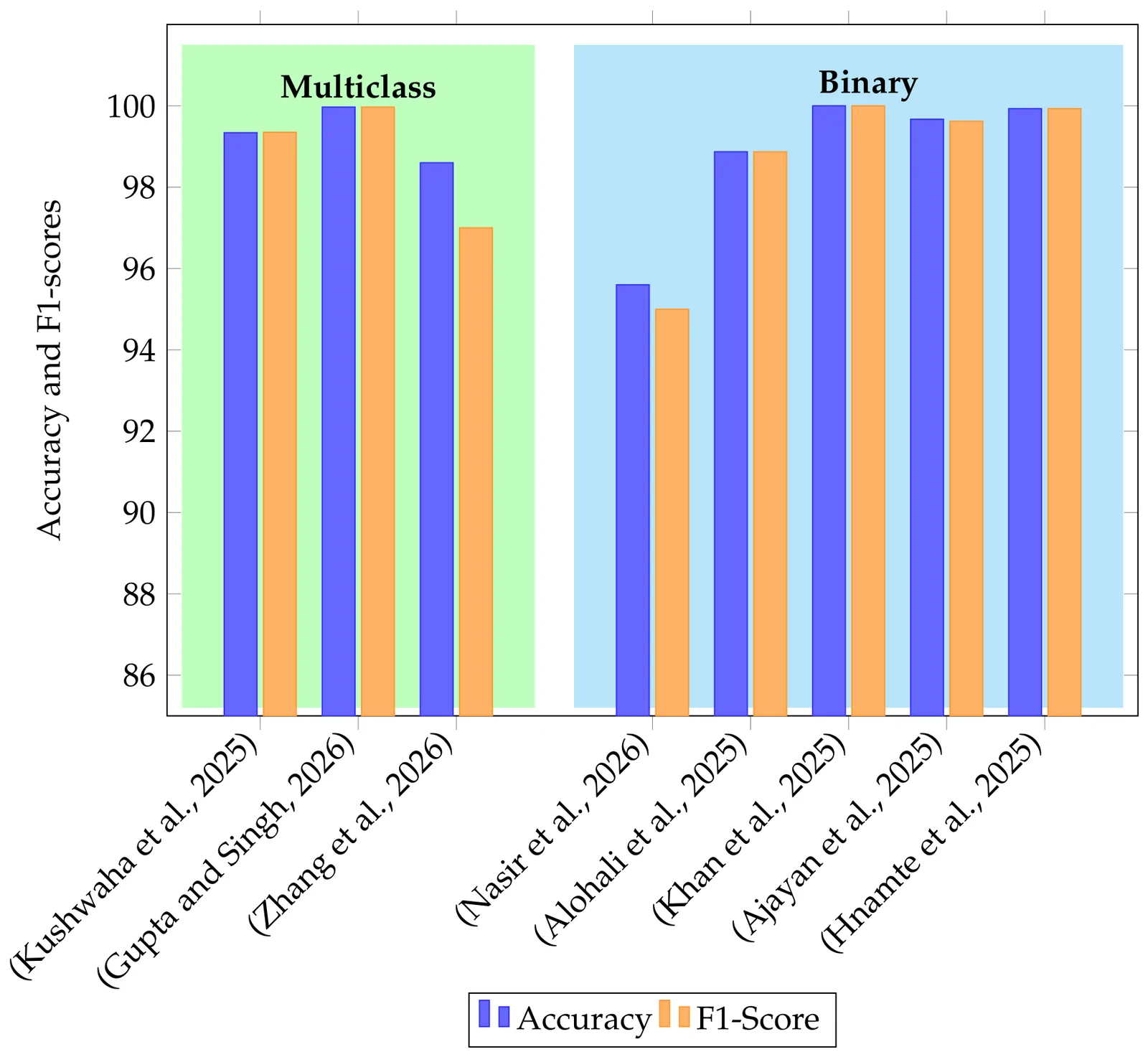
Comparison performance metrics of studies ([39,42,47,50,59,61,62,69]) using the CICIDS2017 dataset.

The accuracy rate and F1-score values obtained by studies using the ToN-IoT dataset are shown in Figure 8, grouped as binary and multiclass. Results from studies containing multiple method results are indicated in the figure with the method name added. Furthermore, results obtained using a subset of this dataset are also shown in the figure with the subset name indicated. Overall, it was concluded that the accuracy rate and F1-score values are in the range of 85–100%. Additionally, binary classification results are seen to be better than multiclass classification results. Similarly, in Figure 9, the accuracy and F1-score values for the CICIDS2017 dataset are presented and grouped as binary and multiclass based on the study. Binary classification was predominantly used for this dataset. Looking at the results, it is observed that the accuracy rate and F1-score values vary between 95% and 100% across the studies.

The accuracy and F1-score values obtained on two different datasets show that the performance of the models can vary under different data distributions and network conditions. The performance differences between the datasets suggest that the models can only provide high accuracy and F1-scores under specific data conditions, and that there may be limitations in terms of generalization under variable and dynamic traffic conditions that may be encountered in real-world network environments. This indicates that the applicability of the current models in real-world conditions such as real-time network traffic, new and previously unseen attack types, and constantly changing network behaviors should be further evaluated.

All studies reviewed in this work are presented with sections outlining the characteristics of the proposed approach, as well as a discussion, comparison with existing methods, and information on open research problems that could be addressed in the future. This provides an overview of the reporting status of these sections in the literature.

Overall, a review of the literature reveals that relevant studies show that IDS models have evolved beyond simply achieving high accuracy rates; they have become explainable, lightweight, privacy-preserving, and real-time operational systems. However, holistic approaches that simultaneously offer explainability, cost-effectiveness, data privacy, and adaptability to different IoT domains remain limited. It is anticipated that future IDS models will fill significant gaps in the literature by integrating continuous learning, federated learning, edge intelligence, large language model-supported explainability, and detailed performance evaluations in real-time network environments.

The results of the proposed study assessment framework are presented in Table 4. Each study reviewed is evaluated across four main dimensions: methodological evaluation, experimental reproducibility evaluation, lightweight evaluation, and XAI evaluation. These sub-scores were calculated according to the predefined criteria described in Section 3, and the overall score was then obtained by summing these component scores. This evaluation provides a comparison of the methodological features, experimental reporting, practical applicability to resource-constrained IoT environments, and explainability practices reported in the studies examined. The calculated sub-scores highlight differences in the extent to which these aspects are addressed, providing additional context for interpreting the performance and XAI-related findings reported in the studies reviewed.

Among the included studies, refs. [33,42,66] achieved the highest overall scores, with scores of 9.0, 8.75, and 8.2, respectively. These studies received relatively high scores, indicating that they addressed a broader range of predefined methodological, reporting, lightweight/deployment, and XAI-related assessment criteria than the other studies reviewed. Ref. [33] achieved the highest possible score, suggesting that it provided the most comprehensive coverage of the assessment dimensions among the included studies. Meanwhile, the scores of [42] and [66] indicate that they have similarly comprehensive methodological and experimental characteristics. Nevertheless, the differences in overall scores should be interpreted as differences in the extent to which the predefined criteria were addressed rather than as a direct ranking of scientific validity or model performance. Generally, the results show that a stronger assessment profile within the reviewed lightweight IoT IDS literature can be achieved through comprehensive methodological reporting, evidence of lightweight applicability, and consideration of explainability.

On average, the included studies achieved an overall score of 60.5%, indicating a moderate level of fulfillment of the predefined assessment criteria across the methodological, experimental, lightweight/deployment, and XAI dimensions. The methodological dimension is found to have been addressed to a substantial extent by most of the reviewed studies, achieving a fulfillment level of 85%. The experimental reproductibility dimension also demonstrated a relatively high fulfillment level of 71%, suggesting that the experimental and technical characteristics of the proposed approaches are generally reported with reasonable completeness. In contrast, the lightweight and deployment dimension achieved a considerably lower fulfillment level of 38.5%. This finding suggests that, despite many studies describing their proposed IDSs as lightweight, there is comparatively little evidence regarding their resource efficiency, computational requirements, and practical deployment on IoT devices. Similarly, the XAI dimension is only 42.5% fulfilled, indicating that explainability-related criteria were addressed less consistently than methodological and reporting criteria. Overall, these findings highlight a clear discrepancy between the methodological evaluation and the practical and explainability-oriented evidence in the existing literature. While the reviewed studies generally provide a substantial amount of methodological and experimental information, there is relatively little evidence to support the lightweight deployment of XAI-based explanations or their reliability.

Compared with recent related reviews, this paper focuses specifically on the intersection of explainability and lightweight design in IoT-based IDSs. Ref. [76] provides a broader systematization of XAI across cybersecurity applications, considering different stakeholders and application objectives, including explanation verification and robustness. Ref. [77] examines AI-based cybersecurity in IoT–edge systems from a systems-oriented perspective, covering methods, datasets, evaluation practices, deployment constraints, and resource-related considerations. This review complements these studies by focusing specifically on IoT-based IDSs and examining explainability and lightweight design together within this context. Beyond identifying the use of XAI techniques, the review distinguishes XAI adoption from the validation of the generated explanations. Similarly, lightweight characteristics are assessed based on the evidence reported in each study. The paper also distinguishes between device-side and computer-side experimental evaluations to provide a clearer picture of practical testing conditions. In addition, a study assessment score is used to evaluate the included studies, and sensitivity analysis is performed to examine the robustness of the main synthesis findings concerning lightweight classification and XAI. Taken together, these elements provide a focused assessment of not only how explainability and lightweight design are incorporated into IoT-based IDSs, but also how these characteristics are validated and supported by the reported evidence.

Future works could explore how a natural language-based justification approach to explaining security risks in IoT automation rules [78] can be evaluated across broader threat scenarios and diverse user profiles. Specifically, the impact of explanations based on real-world threat scenarios, rather than just specification lists, on non-expert users’ ability to understand risks and make safe decisions could be investigated. This would enable the more effective implementation of user-centric and accessible security explanations in IoT systems.

## 6. Conclusions

This study comprehensively reviews the current research literature since 2024 regarding explainable AI-powered intrusion detection systems developed for IoT environments. The studies examined show that AI-based systems achieve high success rates in detecting cyberattacks, and that in recent years, in addition to accuracy performance, criteria such as model explainability, computational cost, data confidentiality, and real-time operation have also become the focus of current research.

Studies in the literature show that deep learning models such as CNN, LSTM, and GRU, and their hybrid designs, are common in intrusion detection, while XAI techniques, especially SHAP and LIME, contribute to the development of IDSs in interpreting model decisions. Furthermore, lightweight models capable of operating on resource-constrained IoT devices are being developed using methods such as knowledge distillation, pruning, feature selection, and attention mechanisms. Additionally, blockchain- and federated learning-based approaches ensure data privacy, while active learning, data balancing, and semi-supervised learning methods offer solutions to the problem of unbalanced datasets.

However, a review of current studies reveals that some significant research gaps remain. Although many proposed models demonstrate high performance on specific datasets, their level of generalizability to different datasets and real-time network traffic has not been sufficiently analyzed. Similarly, explainability remains largely limited to feature importance analyses; the role of the provided explanations in security analysts’ decision-making processes and the reliability of alternative explainability methods have not been comprehensively investigated. Furthermore, there are few studies in the literature that analyze real-world conditions such as energy consumption, memory usage, and latency on resource-constrained devices.

The results of the proposed study assessment framework show that while the levels of methodological evaluation (85%) and experimantal reproducibility (71%) are relatively high in the literature, there are significant shortcomings in the lightweight (38.5%) and XAI (42.5%) dimensions. The overall score of 60.5% indicates that although current studies have made progress in terms of methodology, there are areas that need improvement in terms of applicability in real IoT devices and validation of XAI statements.

In conclusion, current research on IoT-based intrusion detection systems is shifting its focus from high accuracy to smart security approaches that are explainable, lightweight, data-conscious, and adaptable to different network environments. Future studies, integrating explainable artificial intelligence with modern technologies such as federated learning, blockchain, continuous learning, edge computing, and LLMs, and simultaneously performing detailed performance measurements in real-time and across different IoT environments, will significantly contribute to the design of more reliable and feasible next-generation intrusion detection systems.

## Figures and Tables

**Table 1:** Bibliometric analysis of the reviewed papers.

Paper	Author & Year	Title	Quartile	IF	Cite
[27]	Niktabe et al., 2026	Unveiling intruders’ behaviors: explainable AI-based profiling of malicious bot activities in IoT networks	Q2	3.5	-
[28]	Sharma, Shambharkar, 2025	Multi-attention DeepCRNN: an efficient and explainable intrusion detection framework for Internet of Medical Things environments	Q2	3.6	22
[29]	Umair et al., 2025	Knowledge distillation for lightweight and explainable intrusion detection in resource-constrained consumer devices	Q1	9.9	7
[30]	Mittal et al., 2025	A deep learning ensemble approach for malware detection in Internet of Things utilizing Explainable Artificial Intelligence	Q1	9.0	13
[31]	Nadiah et al., 2025	Transformer-based knowledge distillation for explainable intrusion detection system	Q1	6.8	9
[32]	Wahab et al., 2025	An explainable three-way neural network approach for intrusion detection in IoT ecosystem	Q1	7.1	2
[33]	Saiyed, Al-Anbagi, 2026	A Hybrid Explainable AI for DDoS Attacks Detection in Industrial IoT Networks	Q1	8.7	-
[34]	Georgiades, Hussain, 2025	An explainable ai approach for interpretable cross-layer intrusion detection in internet of medical things	Q2	2.9	6
[35]	Turgut, Başarslan, 2025	XBiDeep: A novel explainable artificial intelligence based intrusion detection system for Internet of Medical Things environment	Q1	7.1	4
[36]	Kumar et al., 2024	Blockchain and explainable AI for enhanced decision making in cyber threat detection	Q2	3.5	21
[37]	Ahmad Bilal et al., 2025	Federated learning with Explainable AI for malicious traffic detection in IoT networks	Q2	4.2	-
[38]	Sharma et al., 2024	Explainable artificial intelligence for intrusion detection in IoT networks: A deep learning based approach	Q1	9.4	142
[39]	Khan et al., 2025	A Hybrid Lightweight Deep Learning-Based Intrusion Detection Approach in IoT Utilizing Feature Selection & Explainable Artificial Intelligence	Q2	4.2	3
[40]	Ahmad et al., 2025	An interpretable deep learning framework for intrusion detection in industrial Internet of Things	Q1	7.1	23
[41]	Vishwakarma, Kesswani, 2025	StaEn-IDS: An Explainable Stacking Ensemble Deep Neural Network-Based Intrusion Detection System for IoT	Q2	4.2	2
[42]	Ajayan et al., 2025	DistilXIDS: Efficient, lightweight and explainable Transformer-based language model for real-time network intrusion detection	Q1	6.7	3
[43]	Kumar, Ansari, 2024	An explainable nature-inspired cyber attack detection system in Software-Defined IoT applications	Q1	9.4	23
[44]	Jain et al., 2025	Bridging explainability and security: an XAI-enhanced hybrid deep learning framework for IoT device identification and attack detection	Q2	4.2	6
[45]	Korba et al., 2025	Mitigating IoT botnet attacks: An early-stage explainable network-based anomaly detection approach	Q2	4.2	7
[46]	Javed et al., 2026	DOAL-IDS: Deep Optimized Active Learning Framework for Intrusion Detection in IoT Systems	Q1	6.4	1
[47]	Zhang et al., 2026	FCAST-RF: A Lightweight and Deeply Explainable Intrusion Detection Framework for Edge Gateways	Q2	4.2	-
[48]	Alabbadi, Bajaber, 2025	X-FuseRLSTM: A cross-domain explainable intrusion detection framework in IoT using the attention-guided dual-path feature fusion and residual LSTM	Q2	4.0	9
[49]	Alotaibi et al., 2025	Explainable artificial intelligence in web phishing classification on secure IoT with cloud-based cyber-physical systems	Q1	6.4	14
[50]	Hnamte et al., 2025	A lightweight intrusion detection system using deep convolutional neural network	Q1	5.5	11
[51]	Saheed, Misra, 2025	CPS-IoT-PPDNN: A new explainable privacy preserving DNN for resilient anomaly detection in Cyber-Physical Systems-enabled IoT networks	Q1	5.7	72
[52]	Huang et al., 2026	An explainable and adaptive Internet of Things intrusion detection system supported by Large Language Models	Q1	9.0	2
[53]	Fares, Abd Elaziz, 2025	Explainable tabnet Transformer-based on Google vizier optimizer for anomaly intrusion detection system	Q1	8.0	11
[54]	Hasan, Tasnim, 2025	Real-time explainable IoT security with machine learning and CTGAN-enhanced detection for resource-constrained devices	Q1	5.4	5
[55]	Cerasuolo et al., 2025	Adaptable, incremental, and explainable network intrusion detection systems for internet of things	Q1	9.0	14
[56]	Kalakoti et al., 2025	Federated Learning of Explainable AI (FEDXAI) for deep learning-based intrusion detection in IoT networks	Q2	4.7	9
[57]	Hasnain et al., 2026	An intelligent and explainable intrusion detection framework for Internet of Sensor Things using generalizable optimized active Machine Learning	Q1	7.7	3
[58]	Almadhor et al., 2025	Designing a neuro-symbolic dual-model architecture for explainable and resilient intrusion detection in IoT networks	Q1	4.9	5
[59]	Kushwaha et al., 2025	mFCBF based lightweight intrusion detection system for IoT networks	Q1	5.5	4
[60]	Saiyed et al., 2025	Interactive and explainable optimized learning for DDoS detection in consumer IoT networks	Q1	9.9	14
[61]	Gupta, Singh, 2026	Lightweight ensemble learning based intrusion detection framework with explainable artificial intelligence	Q1	9.0	1
[62]	Alohali et al., 2025	Privacy preserving blockchain integrated explainable artificial intelligence with two tier optimization for cyber threat detection and mitigation in the internet of things	Q1	4.9	-
[63]	Saeed et al., 2025	A novel adaptive hybrid intrusion detection system with lightweight optimization for enhanced security in internet of medical things	Q1	4.9	-
[64]	Sharma et al., 2026	Enhancing IoMT security: a novel blockchain-based anomaly detection framework leveraging explainable AI	Q1	5.5	-
[65]	Aljuhani et al., 2024	An intelligent and explainable SaaS-based intrusion detection system for resource-constrained IoMT	Q1	8.7	33
[66]	Mazroa, 2025	FORT-IDS: a federated, optimized, robust and trustworthy intrusion detection system for IIoT security	Q1	4.9	1
[67]	Albasheer, Agarwal, 2025	OptiXID: An optimized and eXplainable AI framework for intrusion detection in IoT networks	Q1	5.5	-
[68]	Sharma et al., 2025	Interpretable intrusion detection for IoT environments using a self-attention-based explainable AI framework	Q1	4.9	3
[69]	Nasir et al., 2026	HED-ID: an edge-deployable and explainable intrusion detection system optimized via metaheuristic learning	Q1	4.9	1
[70]	Akshya et al., 2025	Explainable AI-driven intrusion detection for securing IoT-enabled autonomous transportation systems	Q1	5.5	4
[71]	Jokic et al., 2025	A convolutional neural network-enhanced attack detection framework with explainable artificial intelligence for internet of things-based metaverse security	Q1	9.0	6
[72]	Wali et al., 2025	Explainable AI and random forest based reliable intrusion detection system	Q1	6.8	21
[73]	Iqbal et al., 2026	A multilayered deep learning framework for cyber attack detection and mitigation in a heterogeneous IIoT ecosystem	Q2	4.4	1
[74]	Zhukabayeva et al., 2025	An Impact-Aware and Taxonomy-Driven Explainable Machine Learning Framework with Edge Computing for Security in Industrial IoT-Cyber Physical Systems	Q1	2.9	1

**Table 2:** Summary of the characteristics and findings of the reviewed studies.

Paper	Handled Problem	Targeted Attacks	Methods Used	Dataset	Environmental Setup	Results
[27]	IoT botnet detection, data imbalance, and lack of explainability	General botnet: Mirai, Gagfyt, IRCBot, Kenjiro, Okiru, and Linux Mirai	XGBoost, LSTM, Bayesian optimization, SHAP	BCCC-Aposemat-IoT-Bot-2024	Python, Keras, TensorFlow, AWS SageMaker, Amazon S3, NTLFlowLyzer	Acc: 99.99%, Prec: 99.99%, Rec: 99.99%, F1: 99.99%
[28]	Lack of data security, intrusion detection, and DL explainability in IoMT	DDoS, DoS, Recon, MQTT, spoofing	Blockchain, CNN, Bi-LSTM/RNN, multi-attention, SHAP, and MA-DeepCRNN	CICIoMT 2024	PyTorch and scikit-learn on Windows 11 Home	Binary 99.49%, 6-class 99.12%, 19-class 98.56%, blockchain 182 TPS
[29]	Need for lightweight and explainable IDSs in resource-constrained IoT devices	Botnet classes, DDoS, DoS, Recon	KD, CNN-LSTM teacher–student model, SHAP	N-BaIoT, CIC-IDS 2023	Keras Tuner, Adam optimizer, Tensor Flow Lite, Snapdrag680 octa-core processor	Acc: 99.88% and 99.87%, F1: 98%, size: 471.67 KB, inference: 34 ms
[30]	IoT malware detection and need for explainability	HorizontalPortScan, Okiru, DDoS, C&C	DLEX-IMD, 1D-CNN, LSTM, ANN, DT, LIME	Aposemat IoT-23, AIoT malware detection	Google Colab, T4 GPU, Python3, TensorFlow, Keras, sklearn	Aposemat IoT-23: Acc: 99.96%, Prec: 99.8%, Rec: 99.9%, F1: 99.9%; AIoT: Acc: 99.57%, F1: 99.5%
[31]	IoT IDS: high computational cost and lack of explainability	DDoS, MITM, Ransomware, Slowloris, MQTT, NMAP, Mirai, Gafgyt	DistillGuard, Transformer-based teacher, SGKD, student model, FGSM, XAI	ToN-IoT, RT-IoT, N-BaIoT	Jupyter, Anaconda, Python, Windows 11, i7-8750H processor, 16 GB RAM, RTX 2070	Student binary Acc: 96.43%, 98.81%, 99.98%; multi-attack Acc: 88.13%, 99.15%, 87.13%, in order of dataset, for teacher and student, memory usage 1.59–80 MB, inference time 0.22–19.81 s
[32]	IoT IDS uncertainty, false alarms, and lack of explainability	DDoS, DoS, Mirai, Gafgyt, Backdoor, Scanning, Ransomware, XSS, Injection, password, C&C, etc.	Ex3WNN, CRT-Fusion, 3WC, GTRS, SHAP, LIME	CICIoT2023, TON-IoT, N-BaIoT, X-IIoTID	Ubuntu 20.04.5 LTS, NVIDIA RTX A5000, Python 3.9, 128 G RAM, Intel(R) Xeon(R) CPU E5-2603	Acc: 99.91%, 99.73%, 99.96%, 98.97%; F1: 99.97%, 99.86%, 99.98%, 99.48%, in order of dataset
[33]	DDoS detection and explainability in IIoT	TCP-SYN, UDP, ICMP, HTTP, TLS flood, TLS renegotiation	HEAD, SHAP, LIME, feedforward DNN, HCI	Edge-IIoTSet, HL-IoT, ToN-IoT	Ubuntu 20.04, i9-10900KF, 64 GB RAM, TensorFlow, SHAP, Streamlit, Raspberry Pi 4	In order of dataset, Acc: 98%, 93%, 96%, pruned-HEAD inference: 1.32 s, 3.23 s, 2.79 s; reduced XAI feature processing time: 940 s, 26,456 s, 10,168 s
[34]	Cross-layer IDS and lack of explainability in IoMT	SlowITe, MalariaDoS, malformed, MiTM data, DoS	K-Means, PCA, SHAP feature selection, NB, KNN, RF, AB, LR, DT, PDP, ALE	ICU-IoT	IoT-Flock tool, MQTT and CoAP traffic, 70:30 stratified split	13 SHAP-selected features out of 52, almost full Prec, Rec, Acc, and F1 in KNN and RF, reduced training and estimation time
[35]	Multiclass attack detection and explainability in IoMT and IoHT IDS	Spoofing, Recon, MQTT, DoS, DDoS, Apache Killer, ARP, CAM, Malaria, Netscan, Rudy, SlowLoris, SlowRead, Smurf	XBiDeep, BiGRU-BiLSTM, RNN, LSTM, GRU comparison, SHAP, LIME	CICIoMT2024, IoMT-TrafficData, ECU-IoHT	Label encoding, Z-score, Keras Tuner, Random Search, Adam optimizer, batch:64, 80:20 split	CICIoMT2024 6-class, 19-class, IoMT-TrafficData, ECU-IoHT Acc: 99.75%, 99.85%, 99.90%, 99.87%
[36]	Data integrity in SHS, insider attack risk, and explainability for IDSs	Binary class attack, DDoS, fingerprinting, insider threat, spoofing, replay attack	Blockchain, C-PoA, attribute-based authentication, PSLSTM, MhSA, SHAP	ToN-IoT, IoT healthcare security	PowerEdge R940xa, two Intel Xeon Gold 6240, 256 GB RAM, eight A100 GPU, TensorFlow 2.5, SHAP 0.39, Python 3.7	ToN-IoT: Acc: 97.27%, Prec: 94%, Rec: 98.5%, F1: 96.20%, AUC: 99.4%, IoT healthcare: Acc: 89.62%, Prec: 99.13%, Rec: 76.18%, F1: 86.15%, AUC: 98.8%
[37]	Data privacy, centralized data collection, and explainability in IoT IDS	DDoS, DoS, MITM, injection, malware, Mirai, spoofing, Recon, Web, BruteForce	FL-XAI model, FedAvg, lightweight DNN, SHAP, LIME	Edge-IIoT, CIC-IoT2023, TII-SSRC-23	Python, TensorFlow 2.x, Keras, SHAP v0.41, LIME, Intel i7 CPU, 16 GB RAM, Colab Tesla K80 with 12 GB RAM	Binary Acc: 99.3%, 99.5%, 99%, multiclass Acc: 97.2%, 98%, 96.5%, in order of dataset, model size: 54.3–60.0 KB, FL communication under 30 MB, SHAP: 0.2 s/instance, LIME: 0.5 s/instance
[38]	Multiclass intrusion detection and explainability in IoT IDS	DoS, Probe, R2L, U2R, generic, exploits, fuzzers	Pearson correlation, DNN, 1D-CNN, 2D-CNN, LIME, SHAP	NSL-KDD, UNSW-NB15, NSL-KDDnew, UNSW-NBnew	Google Colab, TensorFlow, Adam optimizer, lr:0.001, epoch:20, train 60%, val 15%, test 25%	NSL-KDDnew Acc: DNN: 99.3%, tt: 142ms, 1D-CNN: 99.2% tt: 325ms ve 2D-CNN: 99.4%, tt: 340ms, UNSW-NBnew Acc: DNN: 80%, tt: 323ms, 1D-CNN: 80%, tt: 442ms, 2D-CNN: 81%, tt: 455ms
[39]	Lightweight IDSs, feature reduction, and explainability in resource-constrained IoT	Legitimate and malicious RF device, attack types in the datasets used	DL-IID, DNN, BiLSTM, GA feature selection, dynamic quantization, LIME	RF Fingerprinting, CICIDS2017, CICIoMT2024, UNSW-NB15	Google Colab, 12.7 GB RAM, Python 3.10, PyTorch, Adam optimizer, lr:0.0001, batch:128, epoch:50	In order of dataset, Acc: 99.84%, 100%, 97.66%, 99.99%, F1: 99.84%, 100%, 98.81%, 99.99%, size: 108.42 KB
[40]	Multiclass attack detection and explainability in IIoT IDSs	DDoS, DoS, Mirai, Recon, spoofing, injection, XSS, backdoor, malware	XGRU-IDS, ETC feature selection, GRU, SMOTE, standard scaler, SHAP	CICIoT2023	Google Colab Pro Plus, NVIDIA L4 GPU, TensorFlow, Keras, Adam optimizer, lr:0.001, batch:1024, epoch:120	Acc: 97.56%, Prec: 97.41%, Rec: 97.56%, F1: 97.42%, Cohen’s Kappa: 97.36%, prediction time 76 μs/sample
[41]	Ensemble model reliability in IoT IDSs, minority class attacks, and explainability	HTTPFlood, TCPFlood, UDPFlood, BruteForce, DDoS, DoS, Recon, injection, backdoor, XSS, etc.	StaEn-IDS, DIDS, CNN, LSTM, RF meta classifier, LIME, SHAP	CIC-IoT IDS 2022, NF-ToN-IoT, NF-BoT-IoT, NF-UNSW-NB15, NF-CSE-CICIDS2018, NF-UQ-NIDS	Adagrad optimizer, lr:0.01, epoch:50, TFLite, Raspberry Pi 3, PyShark, Ettercap	CIC-IoT IDS 2022: HTTPFlood TCPFlood BruteForce Acc: 100%, in order of other datasets, Acc: 91.58%, 95.82%, 99.49%, 98.44%, 97.72%, TFLite size: 811 KB, inference: 1.5 ms
[42]	DDoS detection, class imbalance, lightweight, real-time, and explainability	DDoS	DistilXIDS, DistilBERT fine-tuning, class-weighted loss, SMOTE, SHAP, LIME	CIC-IDS2017, CIC-DDoS2019, UNSW-NB15	Red Hat Enterprise Linux Server release 7.9, Tesla V100 GPU, Xeon Gold 5120 CPU, 128 GB RAM, PyTorch, Hugging Face, AdamW optimizer, batch:16, epoch:10	In order of dataset, baseline Acc: 99.64%, 99.87%, 98.02%; class weighted Acc: 99.74%, 99.88%, 97.82%; SMOTE Acc: 99.61%, 99.78%, 97.54%; avg latency 8.22–52.03 ms and GPU utilization 37.54%–94.00% for batch size 1-32, avg inference 10.05 ms, 7.79 ms, 7.54 ms
[43]	Lightweight IDSs in SD-IoT, feature selection, and explainability	DoS, DDoS, port scanning, OS fingerprinting, fuzzing	SFOA-LASSO, DT, RF, XGBoost, MLP, SHAP	SD-IoT, CIC-IoT-2023	Python 3.7.9, Scikit-Learn, SHAP, Anaconda, Windows 10, 128 GB RAM, Xeon Gold CPU, two RTX 2080 Ti	SD-IoT device RF Acc: 99.5% binary, 99.49% multiclass, SD-IoT server RF Acc: 99.39% binary, 99.38% multiclass
[44]	IoT device identification, intrusion detection, and explainability	DDoS, DoS, spoofing, Recon, Mirai, backdoor, injection, web-based attacks	CNN-XGBoost, RF feature selection, standard scaler, t-SNE, SHAP	CICIoT2024-DIAD	Python 3.10, Jupyter, TensorFlow 2.11, Scikit-learn 1.3, XGBoost 1.7, i7-1255U CPU, 16 GB RAM, local Windows 11, cloud Linux, NVIDIA A100 GPU	Acc: 99.92%, Prec: 99.62%, Rec: 99.13%, F1: 99.97%, Spec: 99.27%
[45]	Early detection and explainability of IoT botnet C&C traffic	C&C, scan, file download, heartbeat, Mirai, Okiru, Hakai, Torii, Trojan, Muhstik	Semi-supervised anomaly detection, OSVM, IF, LOF, EE, AE, GAN, LIME	Aposemat IoT-23	Hardware, OS and software not specified	OSVM packet-level: Rec: 99.99%, FPR: 1.53%, C&C DR: 99.51%, FPR: 1.09%, delay: ms-level
[46]	Class imbalance, redundant features, limited labeling, and explainability in IoT IDSs	All attack types, binary: normal or attack	VT, ProWRAS, CapsNet, RSA, ROC-Net, MBAL, MARCO-Net, SHAP, LIME	ToN_IoT	Google Colab, Windows 10, Intel i5-7300U, dataset 80:20 split, 10-fold cross-val	ROC-Net Acc: 87%, Prec: 91%, Rec: 85% F1: 87%, MARCO-Net Acc: 90%, Prec: 89%, Rec: 92%, F1: 90%
[47]	Edge gateway IDSs: Class imbalance, lightweight, and explainability	DoS, DDoS, Bot, BruteForce, web attack, etc.	FCAST-RF, PCA, sliding window, LightGBM pruning, RF, adaptive SMOTE, MTE, SHAP	CICIDS2017	Intel i5-9300 CPU, 16 GB RAM, Python 3.8, scikit-learn, imbalanced-learn, SHAP library	Acc: 98.6%, Macro F1: 97%, Macro Prec: 98.3%, Macro Rec: 95.7%, Bot traffic F1: 87.7%, web attack brute force F1: 98.3%
[48]	Cross-domain, class imbalance, and explainability in IoT IDSs	DoS, DDoS, backdoor, injection, MITM, password, Ransomware, Scanning, XSS, Probe, R2L, U2R, Recon, spoofing	Deep encoder, sparse Transformer, feature fusion, PCA, ResLSTM, SHAP, LIME	TON_IoT Network, NSL-KDD, CICIoMT 2024 (19-class), CICIoMT 2024 (6-class)	Python 3.13.2, NVIDIA P100 GPU	In order of datasets, Acc: 99.4%, 99.72%, 97.66%, 98.05%; F1: 99.4%, 99.72%, 97.46%, 98.02%
[49]	Web phishing detection and explainability in CPS or IoT environments	Phishing URLs, legitimate URLs	XAIAOA-WPC, HHO-FS, MHA-LSTM, AOA, LIME	Benchmark phishing URL dataset	Python 3.6.5, i5-8600k, GeForce 1050Ti 4GB, 16 GB RAM, lr:0.01, batch: 5, epoch: 50	Dataset 80:20 split TRAP Acc: 98.92%, TESP Acc: 99.23%, Dataset 70:30 split TRAP Acc: 99.29%, TESP Acc: 99%
[50]	Lightweight and highly successful IDSs in IoT, IoMT, SDN	DDoS, DoS, PortScan, Bot, Web Attack, Infiltration, Heartbleed, BFA, U2R, Recon	LWIDS-DCNN, 1D-CNN, MaxPooling, Adam-Adagrad-Adamax-Nadam-SGD optimizer	CICIDS2017, CICIoMT2024, InSDN	Windows 11 Pro, i5-10600K, RTX 3060 Ti, 8 GB DDR4 RAM, Anaconda, Python 3.9.13, TensorFlow 2.10, batch: 128, epoch: 10	In order of dataset, Acc: 99.93%, 99.7%, 99.97%; F1: 99.93%, 99.68%, 99.96%; inference: 5.8, 5.6, 5.2 μs
[51]	Privacy, explainability, and resilience for IDSs in CPS and IoT networks	DDoS/DoS, MITM, malware, injection, Recon, RDoS, weaponization, tampering, Exfiltration, Crypto randomware	LSTM-AE, DNN, differential privacy, deep SHAP	Edge-IIoTset, X-IIoTID	RTX 4090, i5-8250U, 128 GB RAM, Python 3.12.3, TensorFlow 2.15, Keras 3.7.2, Sklearn 1.4.1	Binary: Acc: 100%, Multiclass Acc: Edge-IIoTset: 99.98%, X-IIoTID: 99.99%
[52]	Labeled data dependency, explainability, and adaptability in IoT IDSs	DoS, Probe, R2L, U2R, DDoS, Recon, Brute Force, Port scan, ICMP flood	EADL-IDS, DCAE, triangle area maps, K-means, silhouette score, LLM, RAG, Facebook AI similarity search	NSL-KDD, CIC-IoT-2023, ACI-IoT-2023	i7-13700H CPU, 32 GB RAM, RTX 4060 8GB GPU, Adam optimizer, lr:1e-3, batch: 32, epoch: 50	NSL-KDD DCAE: DR: 96.7%, F1: 97%, in order of datasets, AUC: 98.61%, 97.14%, 98.59%, EADL-IDS Acc: binary over 95%, multiclass over 85%
[53]	Hyperparameter optimization and explainability for IDSs in IoT	DoS, Probe, U2R, R2L, DDoS, Mirai, Recon, Spoofing, Web, BruteForce, ARP poisoning, NMAP scan	TabNet, Google OSS Vizier, grid search, random search, Bayesian optimization, SHAP	NSL-KDD, CICIoT2023, RT_IoT2022	Dell laptop, i7, 32 GB RAM, NVIDIA GTX 1650Ti, Python 3.12, TensorFlow 2.15, pytorch_tabnet, lr:0.02, batch: 1024	In order of datasets, Binary Acc: 99.9%, 99.62%, 99.16%, Multiclass Acc: 98.29%, 98.43%, 98.1%
[54]	Class imbalance, explainability, and edge distribution in IoT IDSs	DoS, DDoS, Ransomware, XSS, password, MITM, backdoor, injection, scanning, malicious control and operation, spying, data probing, wrong setup	CTGAN, K-means feature creation, LGBM, XGB, DT, KNN, LSTM, LDA, QDA, Adaboost TabNet, SHAP, LIME	DS2OS, ToN_IoT, Weather, Win 10, Modbus, Network	Jetson Nano with Linux OS ve maxwell 128-core GPU, ARM Cortex-A57 CPU	ToN_IoT: Acc: 97.2%, F1: 89.34%, DS2OS: Acc: 99.41%, F1:99.34%, inference: 0.0097–0.5686 s, size: 2.73–1510 kB
[55]	IoT network IDSs: adaptability to different networks, cross-evaluation, CIL and DIL scenarios, and explainability	DoS, DDoS, information gathering, MITM, injection, malware, Mirai, scanning, backdoor, password, Ransomware, BruteForce	2D-CNN, CIL, DIL, FT-Mem, BiC, FT, SHAP and deep SHAP, UMAP	IoT-NID, TON_IoT, Edge-IIoT	Python, PyTorch, NumPy, pandas, SHAP and UMAP library, train: 70% test: 30% split, FACIL framework: 200 epochs	Binary and multiclass test: over F1: 79%, cross-evaluation F1: decrease to 33%, incremental with F1: increase of over 71%, training time FT: 49%-56%, FT-Mem: 46%-54%, BiC: 20%-34% gain
[56]	Privacy and explainability in FL-based IoT IDSs	Mirai and Gafgyt botnet type, ACK, Combo, junk, scan, SYN, TCP, UDP, UDPplain attack type	FedXAI, HFL, FedAvg, LSTM, SHAP, LIME, integrated gradients	N-BaIoT	AMD Threadripper 3960X CPU, 128 GB RAM, RTX 3090 24 GB, Ubuntu 20.04 LTS, Python 3.9, Flower, PyTorch	Binary: Acc: 99.9%, F1: 99.99%, Botnet-type: Acc: 99.28%, F1: 99.54%, Attack-type: Acc: 94.89%, F1: 94.5%
[57]	Class imbalance, low label data, hyperparameter tuning, and explainability in IoT IDSs	Blackhole, flooding, grayhole, TDMA, web attack, BruteForce	ADASYN, LR, LRALE, LRALU, LRSO, LIME, SHAP	IoSTs, CIC-IDS-DS	Hardware, OS and software not specified	IoSTs Acc: LRALE: 92%, LRALU: 91%, LRSO: 84%, CIC-IDS-DS Acc: LRALE: 98%, LRALU: 97%, LRSO: 97%
[58]	Explainability, reliability, and neuro-symbolic decision support in IoT IDSs	DoS, DDoS, theft, Recon	ANN, 1D-CNN, SHAP, LIME, symbolic reasoning	NF-BoT-IoT-V2	Hardware, OS and software not specified, dataset 80:20 split, Adam optimizer	ANN Acc: 98%, Macro F1: 76%, CNN Acc: 98%, Macro F1: 77%, DoS F1: 98%, DDoS F1: 99%, Recon F1: 93%
[59]	High resource consumption, redundant features, and class imbalance in IoT IDS	DoS, DDoS, Patator, Probe, R2L, U2R, Data exfiltration, Recon	mFCBF, K-Means sampling, SVM-SMOTE, LightGBM, XGBoost	CICIDS2017, NSL-KDD, BoT-IoT	Win 10, 8 GB RAM, Intel i5-7500 3.40 GHz CPU, scikit-learn, NumPy, pandas	Acc: CICIDS2017: 99.34%, NSL-KDD: 99.62%, BoT-IoT: 99.75%; CPU: 2.03%, Memory: 1.2%
[60]	High- and low-volume DDoS detection, edge optimization, and explainability in CIoT networks	TCP SYN flood, UDP flood, HTTP flood, TLS flood, Slowloris, Slow HTTP, TCP SYN low, TLS low	ADEPT, CNN and LSTM attention ensemble, DE pruning, min–max quantization, SHAP	ToN-IoT, CICIoT-2023, TTC	i9-10900KF CPU, 64 GB RAM, Python 3.10.9, Keras 2.12, TensorFlow 2.12, Raspberry Pi 4, 4 GB RAM, 1.5GHz 64-bit quad-core Cortex-A72	Acc: TTC: 94%, Testbed-High: 94%, Testbed-Low: 92%, ToN-IoT: 98%, CICIoT-2023: 93%, latency: 0.57 s, throughput: 1775 samp/sec, memory: 746 MB, F1: 93%, SHAP explanation on 1000 samples: 139 s
[61]	Lightweight, multiclass, and explainable IDS design	DoS, DDoS, Port Scan, Brute Force, web attack, XSS, SQL injection, Botnet, Heartbleed, flooding	Feature selection with MI score, PCA, RF, DT, XGBoost, LightGBM, hard voting, SHAP	MSCAD-2022, CICIDS-2017	Win 10, Intel i5 CPU, 8 GB RAM, Python 3.11.1, scikit-learn 1.4.0, Jupyter Notebook	Hard voting Acc: MSCAD: 99.92%, CICIDS: 99.97%, 10-fold cross-val Acc: MSCAD: 99.92%, CICIDS: 99.98%, after PCA processing time MSCAD: 1.17 s–35.71 s and CICIDS2017: 1.26 s–10.85 s, voting ensemble processing time 9.40–12.24 s
[62]	Secure, optimized, and explainable cyber threat detection in IoT	Binary (normal and anomaly)	Blockchain, LSN, POA, A-LSTM-BiGRU, EOA, SHAP	NSL-KDD, CICIDS-2017	Hardware, OS and software not specified, train: 70% test: 30%, 25 epochs, 100,000 samples per dataset	NSL-KDD Acc/F1: 98.34%, CT: 5.38s, CICIDS-2017 Acc/F1: 98.85%, CT: 8.87 s
[63]	Low latency, class imbalance, adaptive threat detection, and generalizability in IoMT IDSs	Spoofing, Recon, DoS, DDoS, ARP spoofing, port scan, OS scan, etc.	SMOTE, RF and permutation feature importance, C4.5, XGBoost, RF, voting ensemble, DQN, C4.5-DQN	CICIoMT2024, WUSTL-EHMS, ECU-IoHT, DF-IoMT, CICIoT2023	i5-8250U CPU, Intel UHD 620 GPU, 24 GB RAM, Python 3.12, scikit-learn 1.6.1, PyTorch 2.7.1, Gym 0.26.2, 80:20 split, 1500 DQN episode, batch: 16, Adam optimizer, lr:0.001	CICIoMT2024: C4.5 Acc: binary: 99.03%, 5-class: 98.55%, 14-class: 99.56%, latency: 9.64 ms, C4.5-DQN binary Acc: 99.2%, avg inference 9.64 ms–25.24 ms, DQN inference 23.35 ms/packet flow, CPU latency under 10 ms
[64]	Blockchain-supported, explainable, and multiclass anomaly detection in IoMT	DDoS, DoS, Recon, MQTT, spoofing, replay, malform data, port scan, ping sweep	Optuna-XGBoost, MI feature selection, min–max normalization, correlation removal, Nik512, ZKP, blockchain, SHAP, LIME	CICIoMT-2024	Win 11, i5-12500HCPU, 16 GB RAM, Intel Iris Xe, Nvidia DGX A100, TensorFlow	Acc: 99.9%, Prec: 99.78%, Rec: 99.89%, F1: 98%, Spec: 99.98%
[65]	Lightweight and explainable IDSs based on SaaS for resource-constrained IoMT	MitM, spoofing, data injection, DDoS, replay, authentication bypass	PSO feature selection, LR, SVM, DT, RF, KNN, ELM, SHAP	WUSTL-EHMS-2020	Apple M1 Pro CPU, 16 GB RAM, macOS Ventura, Jupyter, Anaconda, scikit-learn, scikit-elm, pyswarms	PSO-DT: Acc: 96.56%, Prec: 97.78%, Rec: 98.26%, F1: 98.02%, test time: 0.619 ms
[66]	Cross-domain generalization, adversarial resilience, privacy, and distribution cost in IoT IDSs	DDoS botnet traffic, malicious flows	SMOTE, feature mapping, SHAP, LIME, GNN, CNN, LSTM, FL	DDoS Botnet IoT, UNSW-NB15	i9-13900K CPU, RTX 4090 GPU, 128 GB RAM, Ubuntu 22.04, Python 3.10, PyTorch 2.2.1, Raspberry Pi 4, Jetson Xavier NX, TP-Link HS110 Smart Plug	LR F1:80%, RF F1: 91%, MLP F1: 95%, train-DDoS, test-UNSW F1: 39%, train-UNSW, test-DDoS F1: 99%, FL F1: 96.6%, Pruned CNN int8: latency 47.8 ms, energy 0.42 J, 310 MB peak memory, 72% CPU. TinyCNN int8: latency 21.6 ms, energy 0.18 J, 420 MB, 68% GPU
[67]	Large size, class imbalance, and explainability in IoT IDSs	Analysis, backdoor, DoS, exploits, fuzzers, generic, Recon, Shellcode, Worms, DDoS, malware, MITM, Mirai, web app attacks	ASO feature selection, safe-Level SMOTE, XGBoost, DT, RF, LightGBM, SHAP	NF-UNSW-NB15, CIC-IoT-2023	Hardware, OS and software not specified	UNSW: Acc: 98.4%, Prec: 98.6%, Rec: 98.4%, F1: 98.6%, CIC: Acc: 99.48%, Prec: 99.47%, Rec: 99.48%, F1: 99.47%
[68]	Manual feature selection, explainability, generalizability, and lightweight in IoT IDSs	DoS, DDoS, Recon, theft, keylogging, data exfiltration, service scanning, Mirai, Bashlite, fuzzers, backdoor, Shellcode, Worms	SA-DNN, LFG, CFS and RF feature selection, random oversampling and undersampling, SHAP, LIME	BoT-IoT, N-BaIoT, UNSW-NB15	Google Colab Pro, NVIDIA Tesla T4 GPU, 15.36 GB VRAM, CUDA 12.2, 13.29 GB RAM, 10 run, train: 70%, val: 15%, test: 15%	BoT-IoT: Acc: 99.3%, F1: 99.2%, N-BaIoT: Acc: 99.6%, F1: 99.5%, UNSW-NB15: Acc: 97.9%, F1: 97.5%, latency: under 3 ms, training time: 28 s–44 s and peak memory under 6 GB
[69]	Edge-compatible, explainable, and optimized IDSs	DoS, DDoS, BruteForce, Botnet, web attack, infiltration, injection, backdoor, ransomware, Recon, MITM, XSS, scanning, exploits	S-BiGRU, attention mechanism, GWO, SHAP, quantized edge inference	UNSW-NB15, CICIDS-2017, ToN-IoT, CIC-IDS2018, TON_IoT full	Cloud-like: Intel i7 CPU, RTX 3060 GPU, 16 GB RAM, edge-like: 2 CPU, 8 GB RAM, no GPU, Jetson Nano Raspberry Pi, PyTorch, train: 70%, val: 15%, test: 15% split	In order of datasets, Acc: 92.1%, 95.6%, 93.1%, 94.8%, 93.5%, Cloud Acc: 95.6%, lat: 18 ms, mem: 92 MB, Edge Acc: 93.8%, lat: 22 ms, mem: 115 MB, SHAP explanation: 3–4 ms/instance
[70]	Real-time, explainable, and energy-efficient IDSs for IoT-enabled autonomous vehicle communication	Jamming, data injection, spoofing, unauthorized access, replay	LSTM, RF, SHAP, node isolation procedure	Synthetic, UNSW-NB15	Intel Xeon CPU, 64 GB RAM, RTX 3090 GPU, Python, TensorFlow, scikit-learn, SUMO, OpenStreetMap	Acc: 94.2%, Prec: 92.4%, Rec: 93.5%, F1: 92.9%, lat: 15.2 ms, FPR: 3.2%, energy: 2.1 J, PDR: 98.5%, mem: 320 MB
[71]	Attack detection and attack classification in metaverse IoT networks	DDoS, DoS, Recon, web-based, BruteForce, spoofing, Mirai	CNN, CatBoost, AdaBoost, DAPSO, PSO, VNS, GA, FA, ABC, SCHO, COLSHADE, SHAP	CICIoT2023	Hardware and OS not specified, Python, scikit-learn, 20% stratified sample, 70:30 split, CNN lr:0.0001-0.003, epoch: 10-30, CatBoost lr:0.001–0.1, iter:100–500	Binary CNN-CB-DAPSO Acc: 99.32%, F1: 99.33%, Multi CNN-CB-DAPSO Acc: 87.68%, F1: 86.63%, Binary CNN-AB-DAPSO Acc: 99.28%, F1: 99.30%, Multiclass CNN-AB-DAPSO Acc: 87.49%, F1: 86.55%
[72]	Adverse resilience, reliable prediction, and explainability in IDSs	SSH ve FTP BruteForce, DoS, DDoS, HopSkipJump, ZOO	RF, SHAP, CAM, window-based classifiers, GPT-2 payload analyzer	CSE-CIC IDS 2018, CIC-IoT 23	Hardware and OS not specified, UMNIDS tool, train: 50% test: 50%, train data train: 90% val: 10%, 5-fold CV, GPT-2	CSE-CIC Acc, F1: 99.05%, CIC-IoT Acc: 98.15%, F1: 98.14%, ER CSE-CIC: HSJ: 1.9%, ZOO: 6.2%, ER CIC-IoT: HSJ: 6.4%, ZOO: 5.5%
[73]	High FPR, class imbalance, and edge distribution in IIoT IDSs	DoS, Recon, command injection, backdoor, C&C, ransomware, exfiltration, exploitation, lateral movement, tampering, weaponization	RF feature selection, SMOTE, MLP, RobustScaler, honeypot-based deception, alert generation	WUSTL-IIoT-2021, X-IIoTID-2022	PC: i7-12700 CPU, 16 GB RAM, Raspberry Pi 5: Quad-core ARM CPU, 16 GB RAM, Jetson Xavier NX: 384-core Volta GPU, 6-core ARM CPU, Python 3.10, PyTorch 2.0, CUDA 11.8	X-IIoTID-Acc: PC i7: 99.99%, Pi 5: 99.82%, Jetson: 99.92%, WUSTL-Acc: PC i7: 99.99%, Pi 5: 99.85%, Jetson: 99.94%, lat: under 30 ms, 40 KB parameter memory, total inference memory 88 KB, training time 357.05 s
[74]	Taxonomy-based, explainable, and edge-aware intrusion detection in IIoT and CPS	Flood, Botnet Mirai, Recon, spoofing MITM, injection, backdoor exploits	K-Means, DBSCAN, PCA, RF, XGBoost, MLP, SHAP	Kaggle IoT Intrusion Detection	GPU-based environment, Python, Sklearn, LabelEncoder, OneHotEncoding	Acc: RF: 99.48%, XGBoost: 99.51%, MLP: 99.15%, Silhouette: K-means: 35.19%, DBSCAN: 30.88%

**Table 3:** Comparative evaluation and comparison of the reviewed studies.

Paper	Discussion	Comparison with Prior Studies	Proposed Open Problem	Lightweight	XAI Method	XAI Validation	Strengths	Limitations
[27]	✓	✓	✓	◐ Resource-constrained edge device usage	✓ Gradient SHAP	✗	High success rate, explainability, and resilience to data instability	Real IoT testing, testing with a single dataset
[28]	✓	✓	✓	✗	✓ SHAP	✓ Expert evaluation, usefulness	A combination of high accuracy, explainability, and data integrity	Real-world live IoMT deployment tested with a single dataset
[29]	✓	✓	✓	✓ Model Shrinkage with KD	✓ SHAP	✗	High performance, small model size, short inference time, explainability	Validation on different real-world datasets
[30]	✓	✓	✓	✗	✓ LIME	✗	High performance, ensemble architecture, testing on two datasets, explainability	Real-time testing and exclusive use of LIME
[31]	✓	✓	✓	✓ Student model with SGKD	✓ Gradient contribution heatmap, layer-wise contribution	✗	Lightweight architecture, Transformer-based teacher model, three datasets, adversarial test	In some datasets, the success rate of multi-attacks was lower, comprehensive field testing on actual IoT devices
[32]	✓	✓	✓	✗	✓ SHAP, LIME	✗	Uncertain class assignment, low false-alarm rate, high accuracy, four datasets	Not tested in a real-world environment and the suspicious class requires manual review
[33]	✓	✓	✓	✓ SHAP-based neuron pruning	✓ SHAP, LIME	✓ Instance-based validation, stability and consistency	High performance, fast inference, Raspberry Pi testing, explainability	Real-world testing
[34]	✓	✓	✓	◐ Feature reduction with SHAP	✓ SHAP, PDP, and ALEs	✗	Cross-layer analysis, 13 key features, explainability	Only focus on ICU-IoT dataset
[35]	✓	✓	✓	✗	✓ SHAP, LIME	✗	Three IoMT datasets, balanced and unbalanced datasets, high accuracy, multiclass attack detection	Real-time IoMT or IoHT deployment and edge device testing
[36]	✓	✓	✓	✗	✓ Data integrity with SHAP	✗	Blockchain, PSLSTM and MhSA, two datasets, explainability with SHAP	Low RE in the IoT Healthcare dataset, real-time IoMT system evaluation
[37]	✓	✓	✓	✓ Lightweight DNN and small model size	✓ SHAP, LIME	✓ Results comparison and consistency	Data privacy with FL, three datasets, binary and multiclass testing, explainability	Testing on real IoT edge devices
[38]	✓	✓	✓	◐ Reducing costs through feature reduction	✓ LIME, SHAP	✗	Comparison of DNNs and CNNs, feature reduction, high model performance, local and global explanations	Class imbalance, using XAI outputs for model improvement
[39]	✓	✓	✓	✓ GA and dynamic quantization	✓ LIME	✗	Small model size, high performance, feature reduction using GA, tested on four datasets	Real-world IoT testing: LIME is limited in handling complex feature interactions; SHAP is not used
[40]	✓	✓	✓	◐ GRUs and short extraction time	✓ SHAP	✗	34-class test, high accuracy, feature analysis with ETC, SHAP summary, importance, force plot	Testing on real-world edge and IIoT hardware; low success rates in categories such as DNS spoofing, Recon-OSScan, and Recon-PortScan; tested with a single dataset
[41]	✓	✓	✓	✓ TFLite and Raspberry Pi tests	✓ LIME, SHAP	✗	Stacking ensemble architecture, focus on minority attacks, multiple datasets, edge device testing	Low performance in certain classes, such as scanning; real-time training with federated learning
[42]	✓	✓	✓	✓ DistilBERT-based lightweight Transformer	✓ SHAP, LIME	✓ Results comparison, stability and consistency	Three datasets, high performance, imbalanced class analysis, real-time inference, explainability	The practice of real-time system integration and the debate over its adaptability to other types of attacks
[43]	✓	✓	✓	◐ Eight and nine features	✓ SHAP	✗	Systematic feature selection with SFOA-LASSO, high accuracy, distinction between SD-IoT devices and servers, and additional validation using CIC-IoT-2023	Testing within a real-time SD-IoT network using a single XAI method (SHAP)
[44]	✓	✓	✓	◐ 25 features and hybrid architecture	✓ SHAP	✗	High performance, CNN-XGBoost hybrid architecture, 25 features, explainability	Real edge device deployment and testing, using only SHAP, testing with a single dataset
[45]	✓	✓	✓	◐ Low-latency packet-level detection	✓ Feature selection, surrogate model, permutation importance, LIME	✓ Results comparison and consistency	Training using only benign data, ability to detect unknown bots, multi-traffic representation, low FPR, early C&C detection	Real-time system integration, low precision, and F1-score for Trojan, tested with a single dataset
[46]	✓	✓	✓	◐ VT and cost reduction through active learning	✓ SHAP, LIME	✗	Balancing with ProWRAS, optimization using RSA, MBAL active learning, Cohen’s kappa, Wilcoxon test	Testing with a single dataset, real-time IoT, and edge deployment
[47]	✓	✓	✓	◐ PCA and LightGBM, lightweight RF-based architecture	✓ SHAP	✗	Lightweight CPU-only build, high macro F1, improvements against minority attacks, PCA-SHAP semantic mapping	Tested only on CICIDS2017; real-world edge gateway testing
[48]	✓	✓	✓	◐ Feature reduction with PCA	✓ SHAP, LIME	✗	Cross-domain testing, high accuracy, dual-path feature fusion, ResLSTM, XAI support	Testing in a real IoT edge environment
[49]	✓	✓	✓	✗	✓ LIME	✗	High accuracy, HHO-FS, optimization using AOA, explainability for phishing	Dependence on the quality and quantity of educational data, real CPS tests
[50]	✓	✓	✓	✓ Lightweight 1D-CNN structure	✗	✗	Three datasets, lightweight system, high success rate, low loss, short inference time, analysis of different optimizers	Explainability, real-world edge and IoT device testing
[51]	✓	✓	✓	✗	✓ Deep SHAP	✗	Privacy protection, very high accuracy, two CPS-IoT datasets, explainability, low-dimensional feature representation	Deep learning and XAI resource costs, the need for validation in real CPS and IoT environments, lightweight adaptation
[52]	✓	✓	✓	◐ A lightweight version of DCAE for binary classification	✓ LLM ve RAG explanations	✓ Quantitative explanation quality metric and explanation reliability assessment	Label-free processing, robust binary detection with DCAE, explanations using LLMs and RAG, low latency, three datasets, testing with Raspberry Pi	DCAE requires retraining when traffic changes, resulting in a high computational load on LLM components
[53]	✓	✓	✓	✗	✓ SHAP	✗	Automatic optimization with OSS Vizier, three datasets, binary and multiclass testing, SHAP explainability	OSS Vizier’s black-box architecture, local optima problem, real IoT and edge testing
[54]	✓	✓	✓	✗	✓ SHAP, LIME	✓ Fidelity, stability, consistency, and coherence	CTGAN balancing, edge device testing, multi-model comparison, explainability	Online traffic flow, overfitting, and model collapse risk in GAN-based generation
[55]	✓	✓	✓	◐ Training time reduction with incremental learning	✓ SHAP and deep SHAP, UMAP	✗	Three IoT datasets, cross-evaluation, CIL and DIL analysis, explainability, open code sharing	Decrease in F1 score in cross-dataset testing, real-world edge deployment, and robustness testing
[56]	✓	✓	✓	✗	✓ SHAP, LIME, IG	✓ Fidelity, faithfulness, robustness, sensitivity, and quantitative explanation quality metric	Privacy-preserving FL, collects secure SHAP statements from clients, high success rate	Testing with a single dataset, real-world IoT deployment and testing
[57]	✓	✓	✓	◐ LR-based lightweight design, but no real device testing	✓ LIME, SHAP	✗	Data balancing, active learning, SO optimization, 10-fold cross-validation, *t*-test, two datasets	Lack of CPU, memory, and power consumption; real-world sensor and edge testing
[58]	✓	✓	✓	◐ A lightweight ANN module for resource-constrained devices	✓ SHAP, LIME	✗	ANN-CNN comparison, smart home simulation, symbolic decision support	F1: 0.00 in the Theft class, real-world edge and IoT hardware testing, testing with a single dataset
[59]	✓	✓	✓	✓ Ten features, low CPU and memory usage	✗	✗	Feature reduction using mFCBF, three datasets, high accuracy, low resource consumption	Real IoT and edge device testing, adaptation to changing network conditions, no XAI
[60]	✓	✓	✓	✓ Modeled on resource-constrained CIoT networks	✓ SHAP, risk assessment	✗	High- and low-volume DDoS detection, real-edge testing, low latency, explainability, multiple datasets	Additional CPU and memory load for the one-time optimization phase; SHAP annotations at the global level
[61]	✓	✓	✓	✓ Five components and short processing time with MI ve PCA	✓ SHAP	✗	High accuracy, two datasets, 10-fold cross-validation, feature reduction, class-based SHAP explanations	Real-world edge and IoT device testing, energy efficiency, and low-cost calculations
[62]	✓	✓	✓	✗	✓ SHAP	✗	Blockchain integration, two-stage optimization, two datasets, high success rate	Only binary classification, real IoT and edge testing
[63]	✓	✓	✓	✓ Ten features, low latency for C4.5	✗	✗	Adaptive C4.5 and DQN hybrid architecture, low-latency classification, generalization test using multiple IoMT datasets	DQN additional latency and complexity calculations, explainability, real-world hospital edge testing
[64]	✓	✓	✓	◐ Off-chain storage	✓ SHAP, LIME	✗	Blockchain, multi-attack class, high success rate, XAI support	A single dataset, real-world IoMT device testing
[65]	✓	✓	✓	✓ Feature selection using PSO and a resource-constrained edge approach, low latency	✓ SHAP	✗	Lightweight architecture, low-latency testing, high accuracy, SHAP explainability	Testing with a single dataset, multiclass classification, real-world edge device testing
[66]	✓	✓	✓	✓ Pruning, TinyCNN and TCN	✓ SHAP, LIME	✓ Stability, consistency, quantitative explanation quality metric	Cross-dataset test, leakage-free protocol, FL privacy, adversarial analysis, edge latency, and energy measurement	Vulnerability to DeepFool in the context of adversarial augmentation, more diverse real-world and more heterogeneous FL tests
[67]	✓	✓	✓	✗	✓ SHAP	✗	Data balancing, two datasets, high accuracy, SHAP explainability	Real-world testing, full resilience against adversarial examples
[68]	✓	✓	✓	✓ LFG, compact attention, and low latency	✓ SHAP, LIME	✗	Three datasets, high accuracy, low false-positive rate, explainability, reporting with 10 repetitions	Testing on real IoT and edge devices
[69]	✓	✓	✓	✓ Edge-like low latency and memory usage	✓ SHAP	✗	Attention mechanism, SHAP explainability, multiple datasets, cloud and edge comparison	Edge testing generally involves simulation, real-world testing, and low recall values for minority classes
[70]	✓	✓	✓	◐ Low latency	✓ SHAP	✗	Hybrid architecture, SHAP explainability, low latency, energy, PDR, and scalability measurement	Largely simulation-based; testing of actual autonomous vehicles and edge devices is limited
[71]	✓	✓	✓	◐ The inference time for the optimized two-layer architecture is relatively low	✓ SHAP	✗	CNN and boosting hybrid architecture, optimization, binary and multiclass testing, statistical analysis, SHAP explainability	Testing with a single dataset, feature selection, and real-world metaverse IoT device testing
[72]	✓	✓	✓	✗	✓ SHAP tree explainer module	✓ Credibility assessment module, consistency, quantitative explanation quality metric	Adversarial defense requiring no retraining, low evasion rate, two datasets, SHAP-based reliability check	White-box penetration testing
[73]	✓	✓	✓	✓ Low latency and low memory usage, Pi 5 and Jetson testing	✗	✗	Multi-layered architecture, class balancing, real-edge testing, honeypot, and mitigation layer	Explainability, testing for adversarial robustness
[74]	✓	✓	✓	◐ Hybrid edge–cloud strategy	✓ SHAP	✗	Attack taxonomy, high overall accuracy, clustering analysis using silhouette score, SHAP explainability	Low recall in minority classes, real-world IIoT testing

**Table 4:** Study assessment scores of the included studies.

Study	Methodological	Experimental Reproductibility	Lightweight	XAI	Overall Score
[27]	0.9	1.5	0.5	0.5	3.4
[28]	1.4	2	0	1.5	4.9
[29]	1.2	2.25	1	0.5	4.95
[30]	1.7	1.5	0	0.5	3.7
[31]	2	3	1	0.5	6.5
[32]	2	2.5	0	1	5.5
[33]	2	3	2	2	9
[34]	1.4	1.75	0.5	1	4.65
[35]	2	1.75	0	1	4.75
[36]	1.7	2.75	0	0.5	4.95
[37]	2	2.5	1	2	7.5
[38]	2	1.75	0.5	1	5.25
[39]	2	2.25	1	0.5	5.75
[40]	1.4	2.25	0.5	0.5	4.65
[41]	2	2.5	2	1	7.5
[42]	2	2.75	2	2	8.75
[43]	1.7	2.5	0.5	0.5	5.2
[44]	1.4	2.5	0.5	0.5	4.9
[45]	1.4	0.75	0.5	1.5	4.15
[46]	1.4	1.5	0.5	1	4.4
[47]	1.4	2.5	0.5	0.5	4.9
[48]	2	1.5	0.5	1	5
[49]	1.4	2	0	0.5	3.9
[50]	2	2.75	1	0	5.75
[51]	1.7	2.5	0	0.5	4.7
[52]	2	2.25	1.5	1.5	7.25
[53]	2	2.5	0.5	0.5	5.5
[54]	1.7	2.5	1.5	2	7.7
[55]	2	1.75	0.5	1	5.25
[56]	1.4	2.5	0.5	2	6.4
[57]	1.7	0.5	0.5	1	3.7
[58]	1.4	0.5	0.5	1	3.4
[59]	2	2.75	1	0	5.75
[60]	2	3	2	0.5	7.5
[61]	1.7	2.5	1	0.5	5.7
[62]	1.7	0.5	0	0.5	2.7
[63]	2	2.75	1	0	5.75
[64]	1.4	2.5	0.5	1	5.4
[65]	1.4	2.75	1	0.5	5.65
[66]	1.7	2.5	2	2	8.2
[67]	1.4	0.5	0	0.5	2.4
[68]	2	2.25	1	1	6.25
[69]	2	3	2	0.5	7.5
[70]	1.7	3	0.5	0.5	5.7
[71]	1.4	1.5	0.5	0.5	3.9
[72]	1.7	1.5	0.5	1.5	5.2
[73]	1.7	2.75	2	0	6.45
[74]	1.4	1.5	0.5	0.5	3.9
Avg/Total	1.70/2	2.13/3	0.77/2	0.85/2	5.45/9
Fulfillment Level	85%	71%	38.5%	42.5%	60.50%

## Data Availability

No datasets were generated or analyzed in this study. The study is based solely on the systematic analysis and synthesis of previously published literature.

## Supplementary Materials

The following supporting information can be downloaded at: https://www.mdpi.com/article/10.3390/s26185744/s1, Table S1: PRISMA 2020 checklist.
